# Molecular mechanisms and genetic factors contributing to the developmental dysplasia of the hip

**DOI:** 10.3389/fgene.2024.1413500

**Published:** 2024-08-02

**Authors:** Xiaoming Zhao, Shuai Liu, Zhonghua Yang, Yong Li

**Affiliations:** ^1^ Department of Pediatric Orthopaedics, Shenyang Orthopaedic Hospital, Shenyang, China; ^2^ College of Police Dog Technology, Criminal Investigation Police University of China, Shenyang, China; ^3^ Department of Pediatric Surgery, Shengjing Hospital of China Medical University, Shenyang, China; ^4^ Key Laboratory of Health Ministry for Congenital Malformation, Shengjing Hospital of China Medical University, Shenyang, China

**Keywords:** developmental dysplasia of the hip, susceptibility gene, single-nucleotide polymorphisms, CX3CR1, GDF5

## Abstract

The most prevalent hip disease in neonates is developmental dysplasia of the hip (DDH). A timely and accurate diagnosis is required to provide the most effective treatment for pediatric patients with DDH. Heredity and gene variation have been the subject of increased attention and research worldwide as one of the factors contributing to the pathogenesis of DDH. Genome-wide association studies (GWAS), genome-wide linkage analyses (GWLA), and exome sequencing (ES) have identified variants in numerous genes and single-nucleotide polymorphisms (SNPs) as being associated with susceptibility to DDH in sporadic and DDH family patients. Furthermore, the DDH phenotype can be observed in animal models that exhibit susceptibility genes or loci, including variants in *CX3CR1*, *KANSL1*, and *GDF5*. The dentification of noncoding RNAs and *de novo* gene variants in patients with DDH-related syndrome has enhanced our understanding of the genes implicated in DDH. This article reviews the most recent molecular mechanisms and genetic factors that contribute to DDH.

## Highlights

• Heredity and gene variation was one of the factors leading to the pathogenesis of DDH.
• Variants in coding and non-coding RNAs were related to the pathogenesis of DDH.
• The review on the genetic factors of DDH help to understand DDH-related genetic information contributing to the early screening and diagnosis.

## Introduction

Developmental dysplasia of the hip (DDH) is a disorder characterized by an abnormal relationship between the femoral head and the acetabulum. Signs of DDH include luxation, subluxation, femoral head instability causing it to migrate in and out of the acetabulum, and multiple radiographic abnormalities reflecting inadequate acetabular formation. DDH is the most prevalent congenital defect in newborns, with an estimated incidence of 3–6/1,000 (Loder and Shafer, 2015). DDH alters hip biomechanics and overloads the articular cartilage, leading to early osteoarthritis. Early DDH detection allows for a simpler and more effective treatment. The Pavlik harness is usually the first-line treatment for infants younger than 6 months with DDH. If the DDH diagnosis is delayed, a pelvic or femoral osteotomy must be conducted within the next few years. However, this procedure can lead to lower limb discrepancy, gait instability, postural scoliosis, hip flexion contracture, gait abnormalities and ipsilateral permanent disabilities such as valgus knee (Yang et al., 2019). DDH is the leading cause of total hip replacement in younger populations, with an estimated incidence of 21%–29% (Vaquero-Picado et al., 2019). Therefore, early diagnosis is crucial for treating DDH to achieve a favorable clinical prognosis. Studies have demonstrated that the etiology of DDH is related to risk factors including maternal estrogen and progesterone imbalance, oligohydramnios, breech presentation during delivery, multiple pregnancy, clubfoot, female gender, ligament relaxation surgery, left hip joint, swaddling with lower extremities wrapped tightly together, macrosomia, and high altitude (Woodacre et al., 2016; Zhao et al., 2019). Of note, DDH is also considered to be a familial aggregation disease and typically affects multiple individuals within a family. The risk of developing DDH increased by 12 times in first-degree relatives of DDH patients (Gkiatas et al., 2019), while the risk of developing DDH in second-degree relatives is only 1.7 times higher than in the general population. According to the American Academy of Orthopedic Surgeons guidelines, breech position, family history, and symptoms of hip instability are risk factors that warrant ultrasound-based screening for DDH in infants (Schaeffer et al., 2018). Ultrasound-based screening has been implemented for infants with risk factors for DDH worldwide. DDH screening in pregnancy is exclusively dependent on ultrasound during the middle and third trimesters, as there is no evidence of hip dysplasia in fetuses aborted before 20 weeks of gestation (Dunn, 1976). This indicates that most changes in DDH are detected in the later stages of the intrauterine period. Therefore, the diagnosis and prediction of DDH during pregnancy are of significant importance, especially in the early stages of pregnancy. Given the familial susceptibility of DDH, understanding the genes underlying the pathogenesis of DDH and conducting screening for these genes during pregnancy will significantly enhance the early diagnosis and treatment of DDH.

DDH demonstrates a diverse array of signs and symptoms, ranging from mild ligament relaxation, subluxation, and complete dislocation of the femoral head to deformities characterized by various forms of systemic skeletal dysplasia or other systemic genetic abnormalities that may involve chromosomal, genetic, and epigenetic changes (Kenanidis et al., 2020). Recently, genetic studies have increasingly employed exome sequencing (ES), whole ES (WES), candidate gene association studies, genome-wide association studies (GWAS), and genome-wide linkage analyses (GWLA) due to their higher output and reliability. Advances in molecular analysis and sequencing technology have enabled researchers to conduct an in-depth study of familial inheritance in DDH. Several chromosomal loci that are closely associated with unilateral or bilateral hip dysplasia, such as *CX3CR1*, *GDF5*, and *HOX*, were identified through familial aggregation studies of multiple DDH patients (Feldman et al., 2010; Feldman et al., 2017; Kenanidis et al., 2020) (Table 1).

**TABLE 1:** Gene variants of developmental dysplasia of the hip (DDH) detected in family study.

Gene	Polymorphisms/Variants	Location	Participants	Genomic design	Mode of inheritance	References
*HSPG2*, *ATP2B4*	HSPG2 (c.3328G > T), ATP2B4 (c.2264G > A)	Saudi	Five members of a family including three DDH patients were all affected	WES	Digenic inheritance (incompletely penetrant)	Basit et al. (2017)
*TXNDC3*	rs10250905	Chinese Han population	15 members of seven families were all affected	Taqman assay	ND	Qiao et al. (2017)
*NOTCH2*	c.2355G > C; p.Lys785Asn	Saudi	Eigth members of a family including four DDH patients were all affected	Sanger sequencing,WGS, ES	Autosomal dominant inheritance with incomplete penetrance	Basit et al. (2018)
*BMP2K*	Two new heterozygous mutations (c.1432_1440delCAGCAGCAG corresponding to p.Gln478_480del and c.1440_1441insCAG corresponding to p.Gln480ins) in exon 11	Chinese	Mutations were found to be present in 4/4 of the affected family members, 4/15 of the unaffected family members	WES, Sanger sequencing	ND	Zhao et al. (2017)
*TRPS1,EIF3H, RAD21,UTP23*	Strongest linkage evidence on chromosomes 8q23-24, covering the 2.377 Mb SNP (rs724717-rs720132), the second strongest linkage evidence on chromosomes 12p12, covering the 0.642 Mb SNP (rs1919980, rs763853, and rs725124)	Chinese Han population	Four-generation Chinese family consisting of 22 members (5 DDH cases)	GWLA	ND	Zhang et al. (2020a)
*TENM3*	Glutamine at position 2,665 was changed into proline	American	Four generations of a family with intergenerational transmission, mutation was detected in all affected members	WES	Autosomal dominant inheritance with incomplete penetrance	Feldman et al. (2019)
*KANSL1*	Missense variant (c.C767T; p.S256F)	Chinese	A 3-generation, 12-member family was studied of which four members were diagnosed with DDH	WES	Dominant mode of inheritance	Xu et al. (2022b)
*CX3CR1*	rs3732378	American	72-member, four-generation DDH family, 38 memberswere affected	GWLA, WES, Sanger sequencing	Autosomal dominant inheritance with incomplete penetrance	Feldman et al. (2013)
HOXB homologous gene cluster	rs16949053, rs11866385	American	Multi-generation DDH family of 18 members, nine members were affected	RFLP	Autosomal dominant manner	Feldman et al. (2010)
*LRP1*	Two rare variants, c.5347C > T (p.R1783W); c.6386C > A (p.T2129K)	Chinese	17 patients with DDH from eight families and targeted sequencing on 68 patients with sporadic DDH, two LRP1 mutation sites in two families and seven sporadic cases	WES, Sanger sequencing	ND	Yan et al. (2022)

ND, Not described; RFLP, PCR-restriction fragment length polymorphism; WES, whole exome sequencing; ES, exome sequencing; GWLA, genome-wide linkage analyses; GWAS, genome-wide association studies.

Additionally, the pathogenic potential of family pedigree-screened variants of enhanced susceptibility, including *TXNDC3*, *HOXB9*, *HSPG2*, and *ATP2B4*, has been investigated in a disseminated population through a case-control study (Rouault et al., 2009; Qiao et al., 2017; Xu et al., 2021) (Table 2). Besides, since more and more reports show that DDH is one of the symptoms of the syndrome such as trisomy 21 (Bennet et al., 1982), 18q deletion syndrome (Yu et al., 2022), and Steel syndrome (Frigola et al., 2021), it is particularly necessary to summarize the results of genetic variants for these syndromes, which has demonstrated phenotypic variability and genetic heterogeneity (Yan et al., 2019) (Table 3). These evidences revealed that a wide range of genetic variants during embryonic development can lead to syndrome-associated DDH. In addition, it has recently been recognized that epigenetic modifications such as RNA methylation may also play an important role in the pathogenesis of DDH (Harsanyi et al., 2020). Moreover, due to the similarities between human and animal models (Doncheva et al., 2021), studies on the identification of DDH susceptibility gene variants are promising in animal models, including pigs, rats, mice, and rabbits. This research has the potential to provide novel insights into the pathogenesis of DDH through epigenetic analyses *in vivo* experiments (Kiapour et al., 2018; Ji et al., 2020; Wu et al., 2020; Liu et al., 2021). Therefore, an increasing number of DDH-related genes have been identified. In this paper, we systematically reviewed DDH-related genes and their functions to better understand DDH-related genetic data.

**TABLE 2:** Gene variants of developmental dysplasia of the hip (DDH) in different populations.

Gene/Loci	Polymorphisms/Variants	Population	Type of study	Case and control number	Genomic design/Detection method	Locus	Risk	References
*ASPN*	ASPN D14, ASPN D13	Chinese	CC	370 cases and 445 controls	RFLP	9q22.31	D14 vs. D13 in all DDH patients and controls, OR = 2.03, CI: 1.38–2.99; D14 vs. D13 in female DDH patients and controls, OR = 2.1, CI: 1.34–3.29, *p* < 0.01	Shi et al. (2011)
*ASPN*	copy number loss of ASPN in the 60 kb region of 9q22.31	Japanese	CC	64 cases and 32 controls	High-density oligonucleotide tiling microarray assay	9q22.31	More DDH (9/64) than controls (0/32); *p* = 0.0212	Sekimoto et al. (2017)
*COL1A1*	T-139C, C-106T, C-35T (rs113647555)	Chinese	CC	154 female cases with 180 controls	Sequencing of the COL1A1 gene promoter	17q21.3–q22.1	Χ2 = 9.917, *p* = 0 .0016	Zhao et al. (2013a)
*COL2A1*	COL2A1 (P allele)	Italy	CC	50 cases and 143 controls	RFLP	12q13.11	OR = 3.127,CI = 1.120–8.730, *p* = 0.02	Granchi et al. (2002)
*COL11A2*	rs9277935	Chinese Han population	CC	350 cases and 595 controls	Taqman assay	6p21.32	TT+GT vs. GG (*p* = 0.017); female patients with GG genotype (*p* = 0.006)	Xu et al. (2020)
*CX3CR1*	rs3732378	Turkey	CC	68 cases and 100 controls	Taqman assay	3p21.3	OR = 12, 95% CI = 5.6–25.7 in AA vs. AG/GG(resessive model),OR = 75.5 95% CI = 10–565 in AA vs. AG/GG(dominant model), *p* < 0.0001	Gumus et al. (2021)
*ESR1*	Xba I, PvuII polymorphism	Japanese	CS	64 DDH patients	RFLP	6q25	Homozygosity was related with higher acetabular index, *p* = 0.03/Pvu II pp associated with low centre-edge (*p* = 0 .07)	Yamanaka et al. (2009)
*GDF5*	rs143383	Chinese females	CC	338 cases and 622 controls	Taqman assay	20q11.22	OR = 1.40; 95%CI = 1.11–1.75, *p* = 0.0037. Female DDH OR = 1.46; 95% CI = 1.21 to 1.91, *p* = 0.0053, severity DDH OR = 1.43; 95% CI = 1.11 to 1.85, *p* = 0.0078	Dai et al. (2008)
*GDF5*	rs143383	Slovakia	CS	118 cases	TaqMan assay	20q11.22	statistically significant in female gender (*p* = 0.002), family history (*p* < 0.001), count of pregnancy (*p* = 0.009)	Harsanyi et al. (2021a)
*GDF5*	rs143383	Slovakia	CC	45 cases and 45 controls	TaqMan assay	20q11.22	OR (TT genotype vs. CT+CC) = 1.52, 95% CI: 1.05–2.19, *p* < 0.05	Harsanyi et al. (2021b)
*GDF5*	hypermethylated	Iran	CC	45 cases and 45 controls	DNA methylation evaluated by metabisulfite method	20q11.22	*p* < 0.01	Baghdadi et al. (2019)
*GDF5*	rs143384	United kingdom	CC	770 patients with DDH	GWLA	20q11.22	allele A, OR = 1.44, 95% CI: 1.34–1.56, *p* = 3.55 *10–22	Hatzikotoulas et al. (2018)
*GDF5*	rs143384, rs143383	Caucasian population	CC	239 cases and 239 controls	allelic discrimination technique	20q11.22	rs143383 OR (TT vs. other genotypes) = 1.71, 95% CI: 1.18–2.48, *p* = 0.026; rs143384 OR (TT genotype vs. CT+CC) = 1.71, 95% CI:1.18–2.48 *p* < 0.05	Rouault et al. (2010)
*GDF5*	rs224332, rs224333	Chinese	CC	192 cases and 191 controls	QIAamp blood kit and HapMap project	20q11.22	rs224332 (X2 = 9.33, *p* = 0.001) (recessive mode) and rs224333 (X2 = 10.88, *p* = 0.001) (dominant mode)	Zhao et al. (2013b)
*HOXD9*	rs711819	Chinese Han women	CC	209 cases and 173 controls	RFLP	2q31.1	OR = 1.79, *p* = 0.045	Tian et al. (2012)
*HOXB9*	rs2303486	DDH formation in Chinese female patients	CC	460 cases and 562 controls	Taqman assay	17q21.3	OR = 1.32, 95% CI: 1.02–1.7 and associated with severity, OR = 1.35, 95% CI: 1.01–1.80 (dominant genetic mode), *p* < 0.05	Hao et al. (2014)
*IL6* and TGFβ1	TGFβ1(rs1800470), IL6 (rs1800796)	Caucasian population	CC	28 cases and 20 contros	RFLP	IL6(7p15.3), TGFβ1(19q13.2)	IL6 (rs1800796) OR = 6.2, 95% CI = 1.3–30, *p* = 0.024; TGFβ1 (rs1800470) OR = 13.4,95% CI = 1.6–110, *p* = 0.016	Kolundzic et al. (2011)
*IL6* and TGFβ1	TGFβ1(rs1800470), IL6 (rs1800796)	Croatia	CC	68 cases and 152 controls	RFLP	IL6(7p15.3), TGFβ1(19q13.2)	IL6 (rs1800796) OR = 6.36,95% CI = 2.57–15.7, *p* < 0.01; TGFβ1 (rs1800470) OR = 2.42,95% CI = 1.08–5.43, *p* = 0.032	Cengic et al. (2015)
*IL6* and TGFβ1	TGFβ1(rs1800470), IL6 (rs1800796)	Chinese Han population	CC	373 cases and 1,115 controls, verified in 691 cases and 2027 controls	Sequenom MassARRAY platform	IL6(7p15.3), TGFβ1(19q13.2)	IL6 (rs1800796) OR = 0.84,95% CI = 0.73–0.93 *p* = 0.0228; TGFβ1 (rs1800470) OR = 1.255, 95% CI = 1.11–1.42, *p* = 0.0004	Ma et al. (2017)
*IL6* and TGFβ1	TGFβ1(rs1800470), IL6 (rs1800796)	Turkey	CC	105 cases and 119 controls	Sanger DNA sequencing	IL6(7p15.3), TGFβ1(19q13.2)	No detailed data	Igrek et al. (2021)
*PAPPA2*	rs726252	Chinese Han population	CC	310 cases and 487 controls	Taqman assay	1q24	NPL score of 2.698 (*p* = 0.0156) and LOD score of 2.119	Jia et al. (2012)
*UQCC*	rs6060373	Chinese	CC	755 cases and 944 controls	GWAS	20q11.22	Allele A, OR = 1.18, 95% CI = 1.01–1.38, *p* = 0 .0338	Sun et al. (2015)
*VDR*	Homozygous t allele mutations in the TaqI region of exon 9	United Kingdom	CC	45 cases, 20 patients with PPA and 101 controls	RFLP	12q13.11	*p* = 0.03	Kapoor et al. (2007)
*WISP3*	rs69306665, rs1022313, rs1230345, rs17073268, rs10456877	Chinese	CC	368 cases and 558 controls	RFLP	6q21	OR between 0.71 and 0.77 (*p* < 0.01)	Zhang et al. (2018)
*WIF1*	rs3782499 AA	Chinese Han population	CC	586 cases and 987 controls	Taqman assay	12q14.3	(AG + GG vs. AA) *p* = 4.37 × 10^−5^)	Sun et al. (2019b)
*FRZB*	rs3768842, rs2242040	Chinese	CC	386 cases and 558 controls	Taqman assay	2q32.1	rs3768842 (OR = 1.46,95% CI: 1.1–1.93, *p* = 0.0081) and rs2242040 (OR = 0.65, 95% CI: 0.48–0.89, *p* = 0.0067)	Xu et al. (2021b)
*TXNDC3*	rs10250905	Chinese Han population	CC	984 cases and 2043 controls	Taqman assay	7p14.1	Allele T: OR: 0.786, *p* = 1.53* 10-5; genotype TT: OR: 0.761, *p* = 0.0075	Qiao et al. (2017)
*TBX4*	rs3744438, rs3744448	Chinese Han population	CC	505 cases and 551 controls	Taqman assay	17p23.2	OR = 0.56, 95% CI: 0.32–0.97/related to severity OR = 0.73, 95% CI: 0.55–0.97, *p* < 0.05	Wang et al. (2010)
rs61930502	rs61930502-A	Chinese Han population	CC	386 cases and 500 controls, verified in 574 cases and 569 controls	GWAS		*p* = 2.0 × 10^−4^	Yan et al. (2019)

RFLP, PCR-restriction fragment length polymorphism; PPA, primary protrusion acetabuli. CC: case-control study; CS, cross-sectional study; OR, Odds Ratio; WES, whole exome sequencing; ES, exome sequencing; GWLA, genome-wide linkage analyses; GWAS, genome-wide association studies.

**TABLE 3:** Gene variants of developmental dysplasia of the hip (DDH) detected in syndrome.

Syndrome	Gene	Polymorphisms/Variants	Clinical presentation besides DDH	Location	Participants	Genomic design	Mode of inheritance	References
18q deletion syndrome	*HSPG2*	10.44 Mb deletion (chr18:67562936-78005270del) at 18q22.2q23, HSPG2 mutations (chr1: 22206699, c.2244C > A, exon 17, p.H748Q; chr1: 22157321–22157321, c.11671+154insA, intron)	Developmental delay, cleft palate, bilateral developmental hip dislocation, hypothyroidism, and recurrent fever	China	One patient	NGS and G-banding karyotyping analysis	ND	Yu et al. (2022)
22q11 micro-duplication syndrome	*TBX1*	Novel 437 kb interstitial duplication at 22q11.21	Microcephaly and mild motor delay, left-sided cryptorchidism, developmental dysplasia of the left hip	American	Patient with DDH, her mother and an older sister has no symptom	Affymetrix Genome-Wide Human SNP Array	ND	Weisfeld-Adams et al. (2012)
aEDS	*COL1A1*, *COL1A2*	Total or partial loss of COL1A1 or COL1A2 exon 6	Connective tissue disorder that is characterized by congenital bilateral hip dislocations, severe generalized joint hypermobility, recurrent joint (sub)luxations, and skin hyperextensibility	Caucasian, Nigerian/Maltese, Japanese	12 patients from 10 families, ten with DDH	NGS	Autosomal dominant manner	Ayoub et al. (2020)
NHD	*COL2A1*	c.2014G>T; p.(Gly672Cys)	Mild form of spondyloepiphyseal dysplasia in which progressive arthropathy of the hip joint is a major manifestation	Namaqualand region of northwestern South Africa	23 family members with DDH	WES	ND	Agenbag et al. (2020)
STLS	*COL27A1*	c.2G>A-p.Gly850Arg- and c.3249+1G>T	Diagnosed in children with short stature, radial heads and bilateral hip dislocations incorrigible with surgical reduction, in addition to a characteristic facies, scoliosis, and carpal coalitions	Spain	Two consecutive infants of a closely related couple	WES	ND	Frigola et al. (2021)
CIPA	*NTRK1*	The frameshift (c.1860_1861insT; p.Pro621fs) mutation and missense mutation (C2125 G > T; p.Val709Leu)	Absence of normal responses to painful stimuli, absent or markedly decreased sweating (anhidrosis) and variable degrees of ID, anhidrosis and a lack of temperature sensing, developmental delay, and DDH	Jordan	Seven patients from five families, three with DDH	RFLP	Autosomal recessive disorder	Masri et al. (2020)
Hyperekplexia	*GLRA1*	Heterozygous Arg271Gln missense mutation in exon 6	Exaggerated startle reflex, neonatal hypertonia, and DDH	Japan	Two patients, one with DDH	RFLP	Autosomal dominant or, occasionally, recessive disorder	Kimura et al. (2006)
Pure trisomy of proximal region in the long arm of chromosome 3	ND	Duplication of 3q12-q23	Severe asphyxia, feeding difficulties, hypoglycemia, and thrombocytopenia, prominent forehead, deep set eyes, and DDH	Germany	One patient	FISH analysis	ND	Gamerdinger et al. (2006)
GMS	*WDR73*	Homozygous missense mutation in the WDR73 gene [NM_032856.3, c.287G > A (p. Arg96Lys)]	Microcephaly, infantile onset of central nervous system, and DDH	Saudi Arabia	One patient	WES	Autosomal recessive disorder	Al-Rakan et al. (2018)
Wiedemann-Steiner syndrome	*KMT2A*	Exons 2–10 deletion and *de novo* nonsense mutation, p.Gln 1978*	Short stature, hairy elbows, facial dysmorphism, developmental delay, and DDH	Korea	16 patients, two with DDH	ES	ND	Ko et al. (2016)
ID	*SYNGAP1*	Missense mutation c.509G>A (ENST00000418600); p.Arg170Gln (ENSP00000403636.2)	Global delay, short stature, general hyperexcitability and aggressive behavior, seizures, almond-shaped palpebral fissures, myopathic appearance, an open-mouthed appearance, and DDH	United Kingdom	10 patients, two with DDH	ES	ND	Parker et al. (2015)
CSPSD	*FAR1*	Heterozygous *de novo* pathogenic variant c.1438C>T (p.Arg480Cys)	Generalized tonic seizures with up-rolling eyes and cyanosis, bilateral cataracts, grade 2 spastic Achilles tendon based on the Ashworth scale, mild weakness (4/5) in upper and lower limbs, and clonus in both ankles, scissoring gait with toe walking, and bilateral hip dysplasia	Saudi Arabia	One patient	ES	ND	Almuqbil et al. (2022)
EDS	*B3GALT6*	c.808G > A(p.(G270S)) and c.942G > C(p.(W314C)	Delayed development, excessive range of motion of joints, patent foramen ovale, skin aging, a spinal deformity, mild kyphosis, special face, joint laxity, slender fingers, and widened right hip joint space	Chinese	One patient	Sanger sequencing	ND	Han et al. (2022)
ARC syndrome	*VPS33B*	c.1157A > C (p.His386Pro)	Arthrogryposis, renal dysfunction, cholestasis, and bilateral hip dysplasia	Spain	One patient	ND	ND	Del Brío Castillo et al. (2019)

ND: Not described. RFLP: PCR-restriction fragment length polymorphism. WES: whole exome sequencing. ES: exome sequencing; VPS33B: VPS33B and lysosome associated.

### Genes related to DDH pathogenesis

#### Asporin

Asporin (*ASPN*) gene is located on chromosome 9q22.31 and encodes extracellular matrix (ECM) protein that belongs to the small leucine-rich proteoglycan (SLRP) protein family. Members of the SLRP family are able to bind to other structural components of ECMs, such as collagen, as well as members of the transforming growth factor-beta (*TGFβ*) superfamily that temporarily reside in ECMs. A study of 370 Chinese patients with DDH showed that the protein encoded by ASPN in DDH patients had a higher 14-aspartic acid repeat sequence (*ASPN* D14) in the N-terminal region, indicating that *ASPN* D14 was positively correlated with DDH. The frequency of D13, a common allele encoding 13 aspartic acid repeats, was significantly reduced, suggesting that *ASPN* D13 may have a protective effect (Shi et al., 2011). In addition, copy number variation (CNV) analysis showed that copy number loss of *ASPN* in the 60 kb region of 9q22.31 was found in 9 of 64 patients with DDH (9/64), which was significantly higher than that in the control group (0/32). Given that deletion of copy number in *ASPN* is associated with severe DDH, all of these 9 patients required surgery (Sekimoto et al., 2017). Previous studies have shown that the *ASPN* D14 allele is a risk gene for knee osteoarthritis (OA) and the D13 allele is a protective genetic factor for OA, and recent studies have shown that the ASPN repeat polymorphism is associated with OA, lumbar disc degeneration (LDD), and DDH. Therefore, these results contribute to our understanding of *ASPN* on the risk of DDH genetics (Loughlin, 2011).

#### Bone morphogenetic proteins

Bone morphogenetic proteins (*BMPs*) are extracted from bone tissue, and more than 40 proteins of the BMP family have been identified. *BMP2* has been widely studied for its important role in embryonic development and postnatal organ development, which can induce mesenchymal stem cells (MSCs) to osteogenesis and differentiation into chondrocytes (Bauge et al., 2014). In cell studies of DDH, proliferation of osteoblast cells was accompanied by upregulation of *BMP2* expression (Xu J. et al., 2022). In a recent study, *BMP2* was used for the treatment of DDH. Specifically, poly-ethylene glycol-lactic acid (PELA) electrospun fibrous scaffolds transplanted with *BMP2* were used to repair the acetabular defect of the micropig. Samples taken after 24 weeks showed the appearance of large quantities of chondrocytes and the formation of part of new bone tissue, suggesting that the novel material with BMP2 component has the potential to treat DDH (Wu et al., 2020). However, in a population from Turkey (68 cases, 100 controls), no significant association between BMP2 (rs235768) polymorphism and DDH was found (Gumus et al., 2021).

BMP2-inducible kinase (*BMP2K*) is located on chromosome 4q21.21, belongs to the transforming growth factor-β superfamily. The protein encoded by *BMP2K* is a serine/threonine protein kinase with a nuclear localization signal, and its expression level is increased during BMP2-induced differentiation of mouse osteoblast cell lines and is critical for bone development and osteoblast differentiation. Thus, *BMP2K* variants may affect hip joint development (Zhao et al., 2017). WES was performed on three affected individuals and two unaffected individuals from four generations of the DDH family. Two new heterozygous mutations (c.1432_1440delCAGCAGCAG corresponding to p.Gln478_480del and c.1440_1441insCAG corresponding to p.Gln480ins) in exon 11 of *BMP2K* were found in three affected individuals and their unaffected grandmother, suggesting that the BMP2K variant may be a cause of DDH(31).

#### Genes of collagen

Collagen, the main component of connective tissue, is a marker of early cartilage degeneration and reflects the mechanical properties of the hip joint (Li et al., 2016). A case study of a 2-year-old girl with DDH reported that intraarticular cartilage tissue was found semi-free during surgery and assumed to originate from the femoral head. This accidental discovery prompted the investigators to conduct histopathological examination of the cartilage tissue. They found that cartilage tissue is composed of collagen fibers and chondroid tissue and expresses Collagen Type I (Chen et al., 2018). Collagen Type I is composed of Collagen Type I Alpha 1 Chain (*COL1A1*) and Collagen Type I Alpha 2 Chain (*COL1A2*) proteins encoded by the type I collagen gene. Autosomal dominant mutation in the type I collagen gene can cause osteogenesis imperfecta (Marini et al., 2017). *COL1A1* is located on 17q21.3-q22.1 and encodes the a1 chain of type I collagen, which is also located in the 4 mb region of chromosome 17q21.32 associated with DDH reported by Feldman et al. (Feldman et al., 2010). Although a case-control study in a Caucasian population (239 cases and 239 controls) from western Brittany, France showed that 10 SNPs of *COL1A1* have no association with DDH (Rouault et al., 2009), Chinese scholars detected three variations in the promoter region of *COL1A1* by comparing 154 female DDH cases with 180 control cases, and believed that DDH of Chinese female is associated with a higher overall variation rate of *COL1A1* gene (Zhao et al., 2013a). Bioinformatic analysis in a recent study conducted on 16 susceptibility genes of DDH showed that *ASPN*, *TGFβ1*, dickkopf WNT signaling pathway inhibitor 1 (*DKK1*), Interleukin 6 gene (IL-6), Teneurin transmembrane protein 3 gene (*TENM3*) and Growth differentiation factor 5 (*GDF5)* were significantly co-expressed with *COL1A1* (Yang et al., 2022). As key regulatory genes influencing the formation of collagen fibers, *COL1A1* and Collagen TypeIII Alpha1 Chain (*COL3A1*) were found to be downregulated by comparing the hip joint capsule of DDH and the control tissue using high-throughput sequencing, which was suspected to be one of the factors leading to joint capsule relaxation due to the loss of collagen fibers and fibroblasts (Yang et al., 2022). In addition, in a study of 12 patients with Arthrochalasia Ehlers-Danlos syndrome (aEDS), the majority (10/12) of patients had total or partial loss of *COL1A1* or *COL1A2* exon 6, five of whom underwent hip joint surgery. This finding suggested that *COL1A1* exon loss may be associated with DDH formation (Ayoub et al., 2020). Fluoride intake was found to affect the degree of hip capsule relaxation. In rat animal models of DDH combined toxicity of fluoride, the incidence of DDH increased with increasing fluoride concentration, showing significantly downregulated *COL1A1* and upregulated *COL3A1* in hip capsule and fibroblast, accompanied by increased oxidative stress and apoptosis levels (Zhou et al., 2022).

Collagen Type II is mainly produced by chondrocytes and provides osmotic function and mechanical support to articular cartilage against compressive stress (Feng et al., 2017). Collagen Type II has been reported to be significantly upregulated in the acetabulum of rabbit DDH models (Zhang et al., 2015). The mRNA level of Collagen type II was significantly upregulated in acetabular cartilage of DDH models established in the swaddling position of Wistar rats (Ning et al., 2018). However, other studies compared Collagen expression in acetabular cartilage of patients with DDH and OA and found that Collagen Type II mRNA level was significantly decreased in DDH, indicating dysregulation of Collagen Type II in cartilage lesions (Feng et al., 2017). Collagen Type II Alpha 1 Chain (*COL2A1*) is located on chromosome 12, and the encoded protein is the most abundant structural protein in cartilage. Mutations in the alpha-1 chain are associated with the onset of rare familial OA (Palotie et al., 1989). Sequencing in two patients has reported that the heterozygous missense mutation of *COL2A1* is associated with the Spondylometaphyseal dysplasia (SMD) corner fracture type (Machol et al., 2017). In addition, a heterozygous mutation of *COL2A1* (c.2014G>T; p.(Gly672Cys)) was found by WES in a multigenerational family with progressive hip disease and mild SMD from the Namaqualand region of northwestern South Africa, which was also confirmed in 23 family members with DDH (Agenbag et al., 2020). Donatella Granchi et al. studied 50 healthy subjects and 143 patients undergoing total hip replacement for idiopathic OA or DDH-associated OA with PvuII restriction endonuclease and found that the *COL2A1* allele was significantly correlated with DDH-associated OA. The probability of *COL2A1* containing at least one P allele is increased by 3 times (95% confidence interval (CI), 1.120–8.730) (Agenbag et al., 2020). Since COL2A1 and vitamin D receptor (*VDR*) are adjacent genes at 12q13 that play a role in chondromorphism and bone mineralization, Granchi et al. reported that SNPs of *COL2A1* and *VDR* genes were associated with DDH-associated OA in the Italian population (Granchi et al., 2002), but SNPs of *COL2A1* and *VDR* genes did not appear to play a role in the association with non-syndromic DDH (Rubini et al., 2008).

Collagen type X was significantly higher in DDH than in the control group (Bo et al., 2012; Ning et al., 2018). A study compared the expression of Collagen in acetabular cartilage of patients with DDH and OA, and found that the mRNA level of Collagen Type II was significantly decreased in DDH, while that of Collagen Type X was significantly decreased. It is suggested that the imbalance of collagen may be related to DDH chondropathy (Feng et al., 2017). In addition, a domestic rabbit DDH model was established by inserting a meniscus into the hip joint to simulate limbus inersion after DDH closed reduction, and the acetabular index and Collagen Type X expression were significantly increased in this DDH model. These results suggest that limbus inversion can accelerate cartilage degeneration in DDH and lead to early OA (Liu et al., 2021).

Collagen type XI alpha 2 chain (*COL11A2*) is located on 6p21.32 and the encoded protein is indispensably involved in collagen formation and extracellular matrix construction in cartilage and bone. Collagen type XI and Collagen type II together constitute the main structure of articular cartilage, suggesting that *COL11A1* may play a role in the development of DDH. A case-control candidate gene association study was conducted in 945 Han patients (350 patients with imaging diagnosis of DDH and 595 healthy controls). The expression of *COL11A2* in DDH patients was significantly lower than that in the control group. In addition, the number of DDH patients with rs9277935TT genotype was significantly higher than that with GG genotype. The chondrogenic effect of *COL11A2* was further verified in cell experiments, suggesting that chondrogenesis inhibited by *COL11A2* rs9277935 variant may be a pathogenic effect of DDH (Xu et al., 2020).

Collagen type XXVII alpha 1 chain (*COL27A1*) is located on 9q32, encodes Collagen type XXVII alpha 1 chain and provides support for connective tissue structure, which is expressed in cartilage in the process of endochondral ossification (Mayo et al., 2009). Two consecutive infants of a closely related couple presented with severe abnormalities and were diagnosed with Steel syndrome (STLS), characterized by the characteristic features of dwarfism and dislocation of bilateral hip and radial heads. In both infants, two variant sites in COL27A1 (c.2G>A-p.Gly850Arg- and c.3249+1G>T) were identified by WES (Frigola et al., 2021). This is the first report of a *COL27A1* variant in STLS. Due to the important role of the *COL27A1* variant in STLS, some of the major skeletal features associated with Steel syndrome, such as shortened body length, scoliosis, and altered skull shape, were observed in *COL27A1* knockout mice, although DDH was not present (Gonzaga-Jauregui et al., 2020), so the role of *COL27A1* in human bone development deserves further study. The above evidence suggests that collagen is an important marker in DDH cartilage, suggesting disease development and prognosis of DDH.

#### C-X3-C motif chemokine receptor 1

C-X3-C motif chemokine receptor 1 (*CX3CR1*) is located on 3p21.3 and belongs to the G-protein-coupled transmembrane receptor superfamily. *CX3CR1* is a co-receptor of the human immunodeficiency virus (HIV) and is overexpressed in the lymph nodes of HIV patients (Arenzana-Seisdedos and Parmentier, 2006)and plays an important role in cell adhesion, differentiation and proliferation of mesenchymal cells into chondrocytes or osteoblasts (Sun et al., 2017). Currently, the role of *CX3CR1* in development is receiving increasing attention. For example, the *CX3CR1* bone marrow lineage plays an important role in lymphatic endothelial remodeling during the development of the mammalian cardiovascular system (Cahill et al., 2021). Bioinformatic analysis of DDH susceptibility genes revealed that *CX3CR1* and GDF5 modulate chondrogenesis through the classical Wnt signaling pathway (Yang et al., 2022). Feldman et al. has found the 2.61MB candidate region of chromosome 3 in 72 family members of four generations using GWLA, and then the *CX3CR1* locus (rs3732378) variant in this candidate region was found in four members with severe symptoms using WES. Later, Sanger sequencing revealed that the variant was present in the DNA of all the 14 individuals with DDH symptoms (Feldman et al., 2013). Later, this team observed that in the *CX3CR1* knockout mice had significantly increased acetabular diameter and joint space, and gait defects such as increased stance width and significantly shortened weight bearing stance time, consistent with human OA. These data provided suggestive evidence for the induction role of *CX3CR1* gene in the pathogenesis of DDH (Feldman et al., 2017). In addition, several studies have reported that *CX3CR1* (rs3732378) polymorphism is associated with DDH (Feldman et al., 2013; Basit et al., 2018; Gumus et al., 2021). Studies in the Turkish population showed that *CX3CR1* (rs3732378) polymorphisms increased the risk of DDH by 12-fold in the covert and 75-fold in the overt group compared with the normal population (Gumus et al., 2021). Due to the increasing attention paid to the role of *CX3CR1* in DDH, recent studies have evaluated the methylation status of *CX3CR1* in DDH patients. However, the results showed that there was no significant difference in the methylation status of *CX3CR1* gene between the 45 pairs of DDH patients and the control group, suggesting that DNA methylation of *CX3CR1* is not involved in the pathogenesis of DDH (Nejadhosseinian et al., 2022).

#### Double von Willebrand factor A

Double von Willebrand factor A (*DVWA*), located on chromosome 3p24.3, is a novel gene identified by a Japanese GWAS. It was found to be specifically expressed in cartilage and binds to ß-tubulin to regulate chondrocyte differentiation (Farquharson et al., 1999; Meulenbelt et al., 2009). Polymorphism in *DVWA* (rs7639618 and rs11718863) has been reported to be strongly associated with the risk of OA in Japanese and Chinese patients (Miyamoto et al., 2008). The correlation between *DVWA* SNPs (rs7639618, rs9864422, and rs11718863) and DDH was analyzed in 368 DDH patients and 508 control patients, which showed that *DVWA* did not seem to be a risk factor for DDH etiology in Chinese Han population (Zhu et al., 2011).

#### Estrogen receptor 1

Estrogen receptor 1 (*ESR1*) is located on chromosome 6q25, is a sex steroid associated with the occurrence of osteoarthritis and osteoporosis, which plays a critical role in skeletal muscle homeostasis and motor function (Ikeda et al., 2019; Tang et al., 2021). *ESR1* has been implicated in the pathogenesis and prognosis of DDH. The expression of *ESR1* in the femoral head ligament and joint capsule of DDH patients was significantly higher than that in the control group, as demonstrated by immunohistochemical staining (Desteli et al., 2013). *ESR1* consists of eight exons separated by seven introns. The mot widely studied polymorphic region is the Xba I-restricted fragment length polymorphism in intron 1. In the study of DDH, the Xba I genotype XX of *ESR1* was found to be more common in patients with DDH than genotypes Xx and xx, indicating that genotype XX may be associated with increased risk of DDH (Kapoor et al., 2007). However, a Japanese report showed that SNPs in the Xba I site of *ESR1* were not associated with the risk of DDH (Yamanaka et al., 2009). PvuII is also located in intron 1 of *ESR1*, and premenopausal women who are *ESR1* PP homozygous have lower bone density than non-PP homozygous women (Rauhio et al., 2011). Although the *ESR1* PvuII polymorphism is not associated with the risk of DDH formation, 90% of *ESR1* PP patients had narrow joint space width and low mid-angle after surgery, suggesting that the *ESR1* PvuII polymorphism is associated with poor prognosis of DDH (Yamanaka et al., 2009).

#### Growth differentiation factor 5

*GDF5* is located on 20q11.22 and encodes bone morphogenetic proteins of the vertebrate-only TGF-β superfamily (Storm et al., 1994), which play a key role in bone and synovial joint formation and endochondral osteogenesis (Harsanyi et al., 2020). The *GDF5*-encoded protein is also a key component of the biological pathways involved in the prenatal and postnatal development of synovial joints (Chijimatsu and Saito, 2019). Many reports have shown that mutations in *GDF5* are associated with abnormal bone development in humans (Genovesi et al., 2021; Rubin et al., 2021). At present, *GDF5* is considered as a key pathogenic gene of DDH and has been studied more in DDH (Kiapour et al., 2018), however, the mechanism on *GDF5* inducing to DDH still needs to be further studied. Short-footed mice with *GDF5* inactivated mutation presented as shorter femur, smaller femoral head and neck, and smaller acetabulum. Cis-regulatory structure of *Gdf5* locus in mice scanned and fine mapped with FVB-NJ strain confirmed many enhancers with DDH-associated risk variants in downstream of *GDF5*, and intervention of downstream genes can restore the above damaged key morphological features (Kiapour et al., 2018). Since *GDF5* (SNP rs143383) is associated with susceptibility to OA, the relationship between GDF5 (SNP rs143383) and DDH was studied, which showed that *GDF5* (rs143383) was significantly correlated with DDH. Especially in female DDH and severe DDH (Dai et al., 2008). Harsanyi et al. studied 118 cases of DDH in Slovakia, and the results showed that although *GDF5* (SNP rs143383) was not the only factor contributing to the genetic component of DDH, the polymorphism of this site had an important influence on the pathogenesis of DDH (Harsanyi et al., 2021a). In addition, this team demonstrated that *GDF5* (SNP rs143383) influenced the occurrence of DDH in 45 patients with DDH in Slovakia (Harsanyi et al., 2021b). In addition, case-control studies showed that methylation of the *GDF5* promoter was significantly increased in cartilage samples from patients with DDH (Baghdadi et al., 2019). A GWLA analysis of 770 patients with DDH found that the heritable component of DDH was attributable to common genetic variations, accounting for 55%, and was evenly distributed across the autosomal and X chromosomes. This study also found a robust association between the *GDF5* promoter (SNP rs143384) and DDH (Hatzikotoulas et al., 2018). A French study involving 239 pairs of Caucasian patients with DDH and controls found a significant association between *GDF5* polymorphism (rs143384, rs143383) and DDH in Caucasians (Rouault et al., 2010). A study from China involving 192 DDH and 191 controls found an association between SNPs and DDH at two sites of *GDF5* (rs224332 and rs224333) in Chinese women, suggesting that GDF5 may be a candidate gene for pahogenesis of DDH (Zhao et al., 2013b). As a number of studies have confirmed the genetic association between *GDF5* and DDH, a functional knee cartilage structure for cartilage repair was generated using 3D bioprinted *GDF5* scaffolds combined with bone marrow mesenchymal stem cells (BMSC), which were transplanted into rabbit knee joints to achieve long-term cartilage protection (Sun et al., 2019a). Recent studies have shown that *GDF5* duplication loci can be associated with more than 20 different diseases. Muthuirulan et al. identified two of the most disease-causing variants, the *GDF5* (rs4911178) variant was associated with DDH and the *GDF5* (rs6060369) variant was associated with knee OA. Futher verification in *GROW1* (where GFP5 rs4911178 resides in) enhancer null mouse has shown have clinically relevant DDH phenotypes and alterations to the orientation of the acetabulum (Muthuirulan et al., 2021).

#### Homeobox

The product of the homeobox (*HOX*) gene on chromosome 17 is a transcription factor associated with bone development and plays a role in embryonic limb development (Sauvegarde et al., 2016). Although a case-control study of a Caucasian population (239 cases and 239 controls) from western Brittany, France, showed no association between multiple SNPs of *HOXB9* and DDH (Rouault et al., 2009), a subsequent study showed that DNA extraction from a multi-generation DDH family of 18 members revealed that a 4 Mb region abnormality on chromosome 17q21.32, which contains the entire *HOXB* homologous gene cluster, may be associated with DDH and was confirmed to be inherited in an autosomal dominant manner (Feldman et al., 2010). *HOXB9* is expressed in human embryos at 5–9 weeks, which coincides with the formation of the original gluteal band, and is expressed in the lumbar spine, sacrum, and caudal regions of the embryo. (Chen and Behringer, 2022). *HOXD9* regulates muscle cell differentiation and growth regulation as well as mesenchymal cell differentiation. A study of Chinese Han women with DDH found that *HOXD9* (rs711819) was significantly correlated with DDH (Tian et al., 2012). Another study in the Han population showed that SNP of *HOXD9* (rs2303486) may be associated with DDH formation in female patients (Hao et al., 2014).

#### Heparan sulfate proteoglycan 2 and ATPase plasma membrane Ca^2+^ transporting 4

Heparan sulfate proteoglycan 2 (*HSPG2*) is located on 1p36.12 and encodes a perlecan protein that crosslinks extracellular matrix components and cell surface molecules and is closely associated with musculoskeletal development in mice and humans (Lowe et al., 2014). ATPase plasma membrane Ca^2+^ transporting 4 (*ATP2B4*) is located on 1q32.1 and encodes the plasma membrane calcium transport ATPase 4 enzyme, which is involved in catalyzing ATP hydrolysis and Ca2 transport out of cells.*ATP2B4* has been shown to maintain bone homeostasis in mice and humans by playing a regulatory role in undifferentiated and mature osteoclasts (Kim et al., 2012). Basit et al. excluded the currently known variation of DDH-related genes (*ASPN*,*CX3CR1*, *DKK1*, *GDF5*, *HOXB9*, *HOXD9*, *PAPPA2*,*TGFβ1*) in five members of a Saudi family (including three DDH patients) using WES and found common haplotypes of *HSPG2* and *ATP2B4* gene variants on chromosome 1 with incomplete penetrance. They predicted that *HSPG2* regulates *ATP2B4* expression through several transcription factors *GATA1*, *MAZ*, and *RFX1* (Basit et al., 2017). Based on the above results, Sanger sequencing was performed in 250 sporadic cases of DDH in China and showed that SNPs of *HSPG2*(chr1:22201470), *ATP2B4* (chr1:203682345), and prostaglandin F receptor (*PTGFR*) (chr1:79002214) were not associated with DDH in Chinese Han population (Xu X. et al., 2021). In addition, *HSPG2* mutation has been reported in the first case of 18q deletion syndrome with DDH. This female child not only has the 18q deficiency syndrome such as developmental delay and cleft palate, but also has bilateral developmental hip dislocation, hypothyroidism, and recurrent fever. NGS testing of the child’s blood samples showed a 10 Mb deletion at 18q22.2 to q23 and two mutations of *HSPG2*, and no variation of DDH-related genes was found in the deletion region, suggesting that DDH may be related to HSPG2 mutation (Yu et al., 2022).

#### Interleukin 6 and transforming growth factor beta-1

*IL-6* is located on 7p15.3, is an immunomodulatory cytokine that plays an important role in the pathogenesis of osteoporosis (Kany et al., 2019). Transforming growth factor beta-1 gene (*TGFβ1*) is located on 19q13.1–13.3, is also reported to be a proinflammatory cytokine and plays an important role in the pathogenesis of OA (Tachmazidou et al., 2019). *IL6* and *TGFβ1* have complex biological roles in bone metabolism, calcium and vitamin D levels and other physiological aspects (Eftekhari et al., 2018). As regulators of fibroblasts and perichondrium cells in tendon, the interaction between *IL6* and *TGFβ1* may affect the development of DDH. In Caucasian populations, the CC genotype located in the IL6 promoter region at site 572 (rs1800796) is associated with DDH and its secondary OA, and the “C allele carrier” in the *TGFβ1* signaling sequence is also associated with DDH and its secondary OA (Kolundzic et al., 2011). However, another study of patients with DDH in Slovakia showed no association between *IL6* (rs1800796) and DDH(73). Čengić et al. compared 68 cases of DDH secondary OA with 152 control cases and found that all three genotypes of *TGFβ1* (29T>C) (rs1800470) and *IL-6* 572G>C (rs1800796) polymorphisms were present in the DDH secondary OA group. However, when family history polymorphism was evaluated, the homozygous mutation rates of TGFβ1T>C (rs1800470) and *IL-6* 572G>C (rs1800796) were found to be significantly higher in patients with positive family history than in patients with negative family history (Cengic et al., 2015). Ma et al. confirmed that the SNPs of *TGFβ1* (rs1800470) and the SNPs of *IL6* (rs1800796) were associated with DDH in a Han population (4206 DDH cases), but the SNP of *IL6* (rs1800796) was weakly associated with DDH (Ma et al., 2017). Conversely, a study showed that SNPs of TGFβ1 (rs1800470) and IL6 (rs1800796) were not the main genetic factors causing DDH (Igrek et al., 2021). In addition, polymorphisms in the TGFβ1 gene were significantly associated with hip osteoarthritis in a Croatian population (Kolundzic et al., 2011).

#### LDL receptor related protein 1

LDL receptor related protein 1 (*LRP1*) is located on 12q13.3, encodes a member of the LDL receptor protein family and has high variability. Knockdown of LRP1 inhibited receptor activator of nuclear factor-κB (NFκB) ligand (*RANKL*) induced osteoclast formation and osteoclast differentiation and proliferation in macrophages (Kohara et al., 2020). A case report revealed an *LRP1* variant associated with syndrome in a brother and sister characterized by cardiopulmonary dysfunction, paddle-shaped fingers and toes, and cloudy corneas. *LRP1* knockout mice may also exhibit skeletal abnormalities during the embryonic period and abnormal limb morphology after birth (Mark et al., 2022). A study performed WES on 17 patients with DDH from eight families and targeted sequencing on 68 patients with sporadic DDH, and identified two *LRP1* mutation sites in two families and seven sporadic cases. Missense variants of *LRP1* knockout mice showed a DDH phenotype with acetabular and femoral head malformation in mice, which was milder in heterozygotes and more severe in homozygotes. *In vitro* experiments have confirmed that *LRP1* deficiency leads to decreased autophagy levels and decreased chondrogenesis capacity (Yan et al., 2022).

#### Pappalysin 2

Pappalysin 2 (*PAPPA2*) is located on chromosome 1 (1q24), encodes PAPPA2, a 1,791-residue protein that is highly expressed in the human placenta during the first trimester. *PAPPA2* was originally thought to be a placental-derived circulating protein and was later demonstrated to play a role in fetal embryo development, postnatal bone growth regulation, and reproductive function (Conover et al., 2011). *PAPPA2* is also a protease of IGF-like growth factor binding protein 5 (IGFBP-5) and regulates insulin-like growth factors (IGFs) required for growth. *PAPPA2* was found to be involved in hip joint development by IGF using the developing mouse hip joint. *PAPPA2* specifically lyses IGFBP-5, resulting in the separation of the biological function of IGF1 from the complex containing IGFBP. In the acetabular and femoral head cartilage of PAPPA2 knockout mice, the chondroproliferation-related proteins Col1A1 and IGF1 were significantly decreased, while IGFBP-5 was significantly increased (Chen et al., 2019). At present, the function of *PAPPA2* in fetal development has received more and more attention (Rosenkrantz et al., 2021; Haider et al., 2022), *PAPPA2* was expressed in osteoblasts during fetal development and in differentiated chondrocytes during intrachondral bone formation (Jia et al., 2012), and *PAPPA2* knockout mice showed reduced body length and femur shortening as adults (Conover et al., 2011). Another study found that the pelvic girdle and coccyx were shortened and the shape of the mandible and pelvic girdle were also altered according to geometric morphometric evaluation in *PAPPA2* knockout mice. The influence of this variation on the size and shape of the pelvic girdle may indicate the correlation between *PAPPA2* and DDH (Christians et al., 2013). In addition, a study using GWLA in a Chinese Han family of 24 individuals (5 DDH patients) found a significant association between *PAPPA2* (rs726252) and DDH, and the *PAPPA2* (rs726252) associated with DDH was verified in 310 DDH cases and 487 control cases (Christians et al., 2013). However, another study showed no significant difference of *PAPPA2* (rs726252) in an extended Han population (697 patients with DDH and 707 patients) (Shi et al., 2014). Meanwhile, a study from Slovakia including 45 patients with DDH and 85 controls also showed that the *PAPPA2* (rs726252) was not associated with DDH (Harsanyi et al., 2021b).

#### Substance P

Substance P (*SP*) is a highly conserved member of the tachykinin family and is widely expressed throughout the animal kingdom. As an 11-amino acid neuropeptide that preferentially activates the neurokinin-1 receptor (*NK1R*), it transmits damaging signals to secondary neurons in the spinal cord and brain stem *via* primary afferent fibers (Zieglgansberger, 2019). The contents of *SP* and its receptor *NK-1R* in the serum and synovium of patients with DDH and DDH-associated OA were significantly higher than those of the normal population. These results suggest that *SP* and *NK-1R* may be associated with hip dysfunction and chronic pain perception and may be involved in the progression from DDH to DDH-related OA (Wang et al., 2014). The expression of *SP* in the synovium was significantly upregulated in patients with severe DDH, but no such significant upregulation was observed in patients with moderate DDH. It was considered that the degree of articular cartilage wear would gradually increase, and the cushioning capacity of the cartilage would gradually disappear. During this process, gradually increased afferent stimulation of joint pain would cause the dorsal nerve root to release more *SP* (Li et al., 2019). Wang et al. showed that *SP* is significantly upregulated in the synovial membrane and synovial fluid of DDH, which is involved in the inflammatory process of arthritis by activating the NF-κB pathway (Wang et al., 2015).

#### Teneurin transmembrane protein 3 gene

The cytogenetic location of *TENM3* is 4q34.3–q35.1, encoding teneurin transmembrane protein 3. *TENM3* belongs to the tenogenic protein superfamily and plays an important role in cell aggregation and adhesion. Feldman et al. performed WES in patients with severe DDH in four generations of a family with intergenerational transmission and found that glutamine at position 2,665 of the *TENM3* gene was changed into proline. Meanwhile, *TENM3* knockout mice were found to have delayed development of the left acetabulum and left articular fossa, and matrix metallopeptidase 13 (*MMP13*), which inhibits differentiation, was overexpressed in the mouse bone marrow cells (Feldman et al., 2019). Recently, a study of 250 cases of DDH of Han nationality found that *TENM3* (OMIM * 610083, chr4:183721398) was not associated with DDH (Xu et al., 2021).

#### Ubiquinol-cytochrome c reductase complex chaperone

Ubiquinol-cytochrome c reductase complex chaperone (*UQCC*) is located on 20q11.22, encodes a zinc-binding protein and is an important candidate gene involved in bone and cartilage development (Liu et al., 2010). Currently, *UQCC* is considered to be a susceptibility gene for DDH (Gkiatas et al., 2019). In a GWAS study of 386 patients with DDH and 558 healthy controls in the Chinese population, 12 SNPs of *UQCC* were found to be associated with DDH, and the *UQCC* (rs6060373) polymorphism significantly correlated with DDH was verified in 755 patients with DDH and 944 controls (Sun et al., 2015). However, a study from Turkey (68 DDHS and 100 controls) showed that *UQCC* rs6060373 was not associated with DDH (Gumus et al., 2021).

#### Vitamin D receptor

Vitamin D receptor (*VDR*), adjacent to the *COL2A1* gene, is located on chromosome 12q13.11 and contains 11 exons with a length of approximately 75 kb. The product of *VDR* binds to vitamin binding D to initiate cellular signaling pathways that regulate and control calcium and phosphorus absorption, thereby affecting bone density. Studies have shown that the serum VD of patients with DDH is not different from that of the normal population, but the level of *VDR* is significantly lower than that of the normal population, indicating that reduced *VDR* may affect the prognosis of DDH (Topak et al., 2021). *VDR* polymorphism is associated with OA (Hassan et al., 2022). In the aforementioned study conducted by Donatella Granchi et al., the distribution of the BsmI polymorphism in *VDR* was also found to be different, with homozygous bb frequency being significantly higher in patients with DDH (Granchi et al., 2002). Previous study has suggested that homozygous t allele mutations in the TaqI region of *VDR* exon 9 were probably associated with higher acetabular index of DDH (Kapoor et al., 2007). However, a study from Japan on 64 DDH patients treated with rotational acetabular osteotomy found that rotational ApaI and Taq I gene polymorphism of VDR are not correlated with the formation and prognosis of DDH (Yamanaka et al., 2009). In addition, no significant correlation was found between four SNPs in the *VDR* gene (rs731236, rs1544410, rs7975232, and rs2228570) and DDH in a case-control analysis using 50 DDH patients and controls from the Saudi population (Jawadi et al., 2018).

#### Genes related to WNT

*WNT* is a large L-cysteine-rich family of glycoproteins ranging in size from 39 to 46 kD. The *WNT* pathway is involved in joint and chondrogenic embryonic development and joint formation (Cahill et al., 2021) and is associated with many stages of vertebrate limb development. Molecules in the *WNT* pathway are involved in proximal-distal growth, dorsoventral patterns, and the development and maintenance of cartilage, bone, muscle, and joint (Church and Francis-West, 2002). In the bioinformatic analysis of 16 DDH susceptibility genes, *DKK1*, *FRZB*, and *WISP3* were members of the *WNT* pathway, suggesting that the *WNT* pathway may be involved in the pathogenesis of DDH (Yang et al., 2022). In the study of DDH, the proliferation of osteoblasts and chondrocytes was affected as interference with Wnt family member 1 (*WNT1*), which also affected the expression of GDF5 and *WNT1* inducible signaling pathway protein 2 (*WISP2*), *BMP2*, and *BMP4* (Xu et al., 2022). In the DDH rat model, the differentially expressed genes in the hip cartilage tissues of DDH and healthy control rats were analyzed by microarray. The results showed that *WISP2* was significantly upregulated and the peroxisome proliferator activated receptor gamma (*PPARG*) signaling pathway played an important role in the development and degradation of cartilage in DDH by KEGG analysis. Acetabular cartilage degradation and apoptosis were significantly increased in DDH rats, accompanied by significantly upregulated *WYP-2*. Cellular experiments confirmed that *WYP-2* negatively regulated the expression of PPARG in chondrocytes, suggesting that *WYP-2* may promote the apoptosis of acetabular chondrocytes by regulating the expression of *PPARG* (Ji et al., 2020). The WNT1-inducible signaling pathway protein 3 gene (*WISP3*) plays an important role in chondrogenesis. It regulates the production of type 2 collagen through the IGF signaling pathway, as *WISP3* is essential for physiological postnatal bone growth and cartilage homeostasis (Cui et al., 2007). Zhang et al. proved that five SNPs in *WISP3* (rs69306665, rs1022313, rs1230345, rs17073268, and rs10456877) were associated with the formation of DDH. They also performed haplotype analyses showing that the GGCGG haplotype was associated with a higher risk of DDH, suggesting that *WISP3* should be used as a reliable biomarker (Zhang et al., 2018). Wnt inhibitory factor 1 (*WIF1*), encoded by the *WIF1* gene, is a lipid-binding protein that binds to the Wnt protein and consists of a unique and conserved WNT inhibitory factor (*WIF*) domain and five epidermal growth factor (EGF)-like domains. The *WIF1* protein inhibits WNT signaling and is expressed primarily in the epiphysis and articular cartilage surface and promotes chondrogenesis (Zhang et al., 2022). The expression of *WIF1* in the hip capsule of canine hip dysplasia was significantly lower than that in normal tissues (Todhunter et al., 2019). A comparative study of *WIF1* expression in hip joint tissues of 586 Chinese Han patients with DDH and 987 healthy controls showed that *WIF1* was significantly increased in DDH patients, and the expression of *WIF1* (rs3782499) AA genotype patients was significantly higher than that of GG genotype patients. The results suggested that the polymorphism of *WIF1* gene rs3782499 might be related to DDH formation (Sun et al., 2019b). Frizzled-Related Protein (*FRZB*) is located on 2q32.1, is an antagonist of the Wnt signaling pathway. *FRZB* is extremely important for chondroblast differentiation of stem cells, and loss of cartilage integrity was observed in *FRZB* -knockout mice (Lories et al., 2007). Xu et al. found a high expression of *FRZB,* which regulates integrin to affect cell adhesion pathways and cell diffusion in DDH, and the rs3768842 and rs2242040 genotypes of FRZB were significantly correlated with DDH (Xu R. et al., 2021).

#### TXNDC3

NME/NM23 family member 8 (*TXNDC3*) is located on 7p14.1, is associated with bone mineral density, chondrocytes, and OA (Yerges-Armstrong et al., 2014). A significant susceptibility of the *TXNDC3* SNP with OA was confirmed in Caucasian and Chinese females (Shi et al., 2008; Machol et al., 2017). A study comparing 984 DDH patients with 2043 healthy controls from a Han population identified a *TXNDC3* rs10250905 missense mutation in 15 members of seven families, confirming an association between the *TXNDC3* rs10250905 and DDH (Qiao et al., 2017).

#### Gene variants in fewer studies and syndromes related to DDH

A case report reports an 8-month-old male child with DDH, microcephaly, mild motor retardation, left undescended testis, and patent ductus arteriosus. CNV of Affymetrix SNP array data showed a 437 kb microduplication on 22q11.21 involved in T-box transcription factor 1 (*TBX1*), catechol-O-methyltransferase (*COMT*), septin 5 (*SEPT5*), G protein subunit beta 1 like (*GNB1L*), thioredoxin reductase 2 (*TXNRD2*) and glycoprotein Ib plateletsubunit beta (*GP1BB*) (Weisfeld-Adams et al., 2012), which have not been reported to be associated with DDH. T-box genes, originally discovered in mice, are highly conserved in vertebrates and play a central role in posterior mesoderm formation and axial development (Schulte-Merker et al., 1994). Loss of T*bx1* results in a bone phenotype similar to cranial dysplasia (Funato et al., 2015). *Tbx4* and *Tbx5* are key regulators of interspecies limb growth and identification during embryonic development (Tickle, 2015). Currently, it has been reported that *TBX4* rs3744438 was not correlated with DDH in Han population (505 children with DDH and 551 controls), while *TBX4* (rs3744448) was significantly correlated with male DDH. Stratified analysis showed that TBX4 (rs3744448) was associated with hip dislocation, but not with subluxation or hip instability (Wang et al., 2010).

A study aiming at exploring new genes associated with DDH using GWAS in a Chinese Han cohort consisting of 386 DDH cases and 500 healthy controls has identified the minor allele rs61930502-A, and there is no gene located around rs61930502 either 20 kb upstream or 20 kb downstream. This intronic SNP tended to prevent DDH in a dominant manner and in addition, was also verified in another Chinese Han cohort consisting of 574 DDH patients and 569 healthy controls (Yan et al., 2019). Meanwhile, protein-protein interaction analysis also revealed that showed the most direct protein interacted with 409 DDH-associated proteins was heat shock 70 kDa protein 8 (*HSPA8*) in this study, indicating its possible role in.pathogenesis of DDH (Yan et al., 2019).

Zhang et al. performed GWLA on a four-generation Chinese family consisting of 24 members (5 DDH cases) and found the strongest linkage evidence on chromosomes 8q23-24, covering the 2.377 Mb SNP (rs724717-rs720132), which resided four genes: transcriptional repressor GATA binding 1 (*TRPS1*), eukaryotic translation initiation factor 3 subunit H (*EIF3H*), RAD21 cohesin complex component (*RAD21*) and rRNA-binding ribosome biosynthesis protein (*UTP23*). Variations in TRPS1 and/or TRPS2 may cause Trichorhinophalangeal Syndrome, which is characterized by fine, sparse, discolored, and slow-growing hair, malnourished nails, small breasts, short stature, short feet, short fingers and DDH (Maas et al., 1993). RAD21 is also involved in the pathogenesis of Trichorhinophalangeal Syndrome because of its nearby location of *TRPS2* (Maas et al., 1993). *EIF3H* and *UTP23* have not been reported to be associated with DDH. In addition, three consecutive markers (rs1919980, rs763853, and rs725124) showed on chromosome 12p12 was 0.642 Mb, in which the only known gene was *SOX5* (Zhang et al., 2020).

Sanger sequencing of a DDH family from Saudi Arabia showed that no known variants in DDH genes (*CX3CR1*, *ASPN*, *HOXB9*, *HOXD9*, *DKK1*, *GDF5*, *PAPPA2*, *TGFB1*, and *UFSP2*). Instead, common homozygous regions on chromosomes 15q13.3 and 19p13.2 were identified and their locations were rs17228178-rs1534200 and rs466123-rs2112461, respectively. Notch receptor 2, chromodomain helicase DNA binding protein 7, DEAH-box helicase 36, pericentrin, and DNA polymerase epsilon, catalytic subunit were detected as genes or gene promoters in the above regions, but no DDH correlation was reported for these genes. In addition, the copy number of chr6p21.32 (chr6:33 053 906-33 069 893) was increased by approximately 15 kb in all affected individuals, and the sixth exon of HLA-DB1 was found in this region. The 6p21.32 (rs6905837) SNP is currently considered to be a novel gene associated with bone mineral density (Liu et al., 2020), but HLA-DB1 is not currently considered to be a pathogenic gene for DDH because partial gains in this region have also been found in non-diseased siblings of this family (Basit et al., 2018). Missense variant in *KANSL1* (c.C767T; p.S256F) was identified as the pathogenic cause of DDH through ES, which was a novel variant co-inherited by all severely affected individuals in A 3-generation, 12-member family. Further study using CRISPR/Cas9 technology to construct *KANSL1* mutant mouse has presented diminished acetabular chondrocytes and defects in acetabular cartilage (Xu et al., 2022)

Notably, DDH is increasingly being reported as one of the features of the syndrome, and additional gene variants were detected that may be associated with DDH besides the above-mentioned variants in *HSPG2* (Yu et al., 2022), *TBX1* (Weisfeld-Adams et al., 2012), *COL1A1, COL1A2* (Ayoub et al., 2020), *COL2A1* (Agenbag et al., 2020), and *COL27A1* (Frigola et al., 2021) (Table 2; Table 3). Frameshift (c.1860_1861insT; p.Pro621fs) and missense variants (C2125 G > T; p.Val709Leu) of *NTRK1* have been reported in patients from Jordan with congenital insensitivity to pain with anhidrosis who were complicated with DDH (3/7) (Masri et al., 2020). A heterozygous missense mutation in exon 6 of the alpha 1 subunit of the glycine receptor gene (*GLRA1*) (Arg271Gln) has been reported in two Japanese hyperekplexia patients, one of whom was diagnosed with hip dislocation (Kimura et al., 2006). The *WDR73* homozygous missense mutation [NM_032856.3, c.287G > A (p. Arg96Lys)] was detected through WES in a patient with Galloway-Mowat syndrome. At 22 months of age, this patient exhibited microcephaly, infantile onset of central nervous system abnormalities, and the right hip demonstrated features of DDH with a shallow acetabular roof (Al-Rakan et al., 2018). Although DDH has been classified as one of the musculoskeletal manifestations of the Wiedemann-Steiner syndrome, it has only been described in two Wiedemann-Steiner syndrome patients with exons 2–10 deletion and *de novo* nonsense mutation, p.Gln 1978* of *KMT2A* (Ko et al., 2016). In two of the ten patients with intellectual disability, a *de novo* missense mutation c.509G>A (ENST00000418600); p.Arg170Gln (ENSP00000403636.2) of *SYNGAP1* was confirmed through ES. This mutation was also associated with unilateral DDH (Parker et al., 2015). Mohammed et al. have reported the first case of a *de novo* heterozygous variant in *FAR1* presenting as cataracts, spastic paraparesis, and speech delay (CSPSD) in the Middle Eastern region. This variant was identified through ES analysis, and it was also the second case of CSPSD with DDH (Almuqbil et al., 2022). Besides, c.808G > A(p.(G270S)) and c.942G > C(p.(W314C) of *B3GALT6* were detected using Sanger sequencing in an Ehlers-Danlos syndrome (EDS) patient with widened right hip joint space (Han et al., 2022). Moreover, the patient with a partial trisomy 3q12-q23 *de novo* who could not be diagnosed as dup(3q) syndrome presented with mild mental retardation, postnatal growth retardation, facial dysmorphisms, and DDH(140). *VPS33B* (c.1157A > C (p.His386Pro)) were detected in a spainish patient of arthrogryposis, renal dysfunction, and cholestasis syndrome with bilateral DDH (Del Brío Castillo et al., 2019).

#### Non-coding RNAs

In the study of the correlation between the FRZB gene and DDH, upstream miRNAs regulating FRZB expression were found in the synovial fluid of patients with DDH. The expression of multiple miRNAs was significantly downregulated, among which miRNA-454 was the most significantly downregulated, and luciferase experiments confirmed that miRNA-454 could lead to the targeted inhibition of FRZB, suggesting that downregulated miR-454 upregulated the expression of FRZB and may play a role in promoting chondrogenesis in DDH joint tissues (Xu et al., 2021).

Cheng et al. compared the hip cartilage of DDH patients and control cases by whole transcriptome sequencing and identified 186 differential miRNA-targeted 175 DDH candidate genes from 1833 differential mRNAs, such as von Willebrand factor A domain containing 1, transmembrane protein 119 and signal peptide, CUB domain and EGF like domain containing 3. Gene ontology (GO) enrichment analysis detected 111 candidate terms for DDH such as skeletal system morphogenesis and skeletal phylogeny. KEGG pathway enrichment analysis identified 14 candidate DDH pathways, such as Hedgehog signaling pathway and Wnt signaling pathway (Cheng et al., 2021). The involvement of DDH-related miRNAs in the screening of DDH differential genes is conducive to further study of the pathogenesis of DDH.

Liu et al. identified 18 differentially-expressed miRNAs using the DDH rabbit model by high-throughput sequencing, and verified that the downregulation of miR-129-5p promoted the expression of its target gene *GDF11*, thus inducing the phosphorylation of *SMAD3*. This pathway can inhibit chondrocyte proliferation and cycling and reduce chondrocyte mineralization nodules, which may be associated with DDH-associated ossification of the acetabular roof (Liu et al., 2020). According to the above sequencing data, members of the same research group verified the low level of miR-1-3p in the acetabular roof cartilage in the rabbit DDH model, and verified the targeting relationship between miR-1-3p and SOX9 at the cellular level. With the silencing of miR-1-3p, *SOX9* expression was upregulated. Genes associated with endochondral osteogenesis, such as *RUNX2* and collagen X, were downregulated (Ding et al., 2021), and the reduction of mineralized nodules produced by cartilage suggested that miR-1-3p may be the cause of abnormal ossification in acetabular cartilage in DDH animals.

In addition, changes in long non-coding RNAs (lncRNAs) were also involved in the pathogenesis of DDH. lncRNA-H19 was downregulated in patients with DDH and in rat models. It has been found that lncRNA-H19 can inhibit DDH cartilage degeneration through competing endogenous RNAs (ceRNAs) mechanism competitive with miR-483e5p combined with Dusp5 in chondrocytes mimicking intermittent cyclic mechanical stress (ICMS) cell force (Wang et al., 2020). Experiments confirmed that lncRNA H19 played a positive role in inhibiting cartilage degeneration.

## Discussion

Due to the diversity of clinical symptoms of DDH ranging from subtle characteristics of dysplastic hips, such as shallowness and underdevelopment of the acetabulum, to grossly deformed acetabulum, which may either be subluxated or dislocated, there is an absence of general official clinical consensus on the screening for DDH. Early diagnosis and management of DDH have been demonstrated to significantly improve treatment efficacy and prevent permanent disability successfully (Zhu et al., 2019). Ultrasound is considered the most reliable examination to screen for DDH in children under 3 years of age, but may not detect DDH in those with smaller lesions. In patients with mild hip instability detected by ultrasound at birth, 96% of the pathologic changes resolved spontaneously within the first 6 weeks after birth (Vaquero-Picado et al., 2019). Accurate imaging and clinical examination that are guided by genetic background knowledge will significantly facilitate the diagnosis and treatment process. Therefore, understanding the genetic basis of the pathogenesis of DDH and conducting early genetic screening may significantly achieve accurate diagnosis and early implementation of correct treatment for DDH and affect the prognosis of DDH.

At present, the genetic basis of DDH is widely acknowledged and proven by familial heredity, and significant progress has been made in the research on the molecular mechanism of DDH. A considerable number of DDH-related genes have been screened due to the extensive application of GWAS, GWLA, and WES. Despite the ongoing debate regarding the potential association of certain genes with DDH, it is unfeasible to disregard the genetic factors that influence DDH. Currently, among the two types of genetic modes of DDH being considered, type I DDH is a multifactorial genetic disease with variable phenotypes and multiple candidate genes including noncoding RNAs (Feldman et al., 2010). Interestingly, there are relatively more studies on DDH-related SNPs than CNVs in Type I DDH, although it has been reported that the DNA sequence diversity within the human genome is more influenced by CNVs than SNPs. Hence, CNVs can be utilized to study DDH and may contribute to the etiological understanding of the genetic basis of DDH in the future (Zamborsky et al., 2019). Notably, single gene variants detected in humans that have been mutated in animal models, such as *CX3CR1*, *GFP5* (rs4911178 (G → A) locate in *GROW1*, a regulatory enhancer active in humans and mice), and *KANSL1*, have been demonstrated to have potential effects on the DDH phenotype (Feldman et al., 2017; Gumus et al., 2021; Xu et al., 2022). Additionally, SNPs of *GDF5* and *TGFβ1* have been demonstrated to be related to DDH in various races (Dai et al., 2008; Rouault et al., 2010; Zhao et al., 2013b; Hatzikotoulas et al., 2018; Sun et al., 2019a; Baghdadi et al., 2019; Harsanyi et al., 2021a; Harsanyi et al., 2021b; Muthuirulan et al., 2021). Nevertheless, it is important to acknowledge that the results were inconsistent across diverse populations. Despite the fact that gene variants such as *ESR1*, *Hoxb9*, *IL6*, *PAPPA2*, *UQCC*, and VDR were associated with DDH, other case-control studies did not report a significant difference (Granchi et al., 2002; Kapoor et al., 2007; Rouault et al., 2009; Yamanaka et al., 2009; Kolundzic et al., 2011; Jia et al., 2012; Christians et al., 2013; Hao et al., 2014; Jawadi et al., 2018; Feldman et al., 2019; Harsanyi et al., 2021b; Gumus et al., 2021).

**FIGURE 1:**
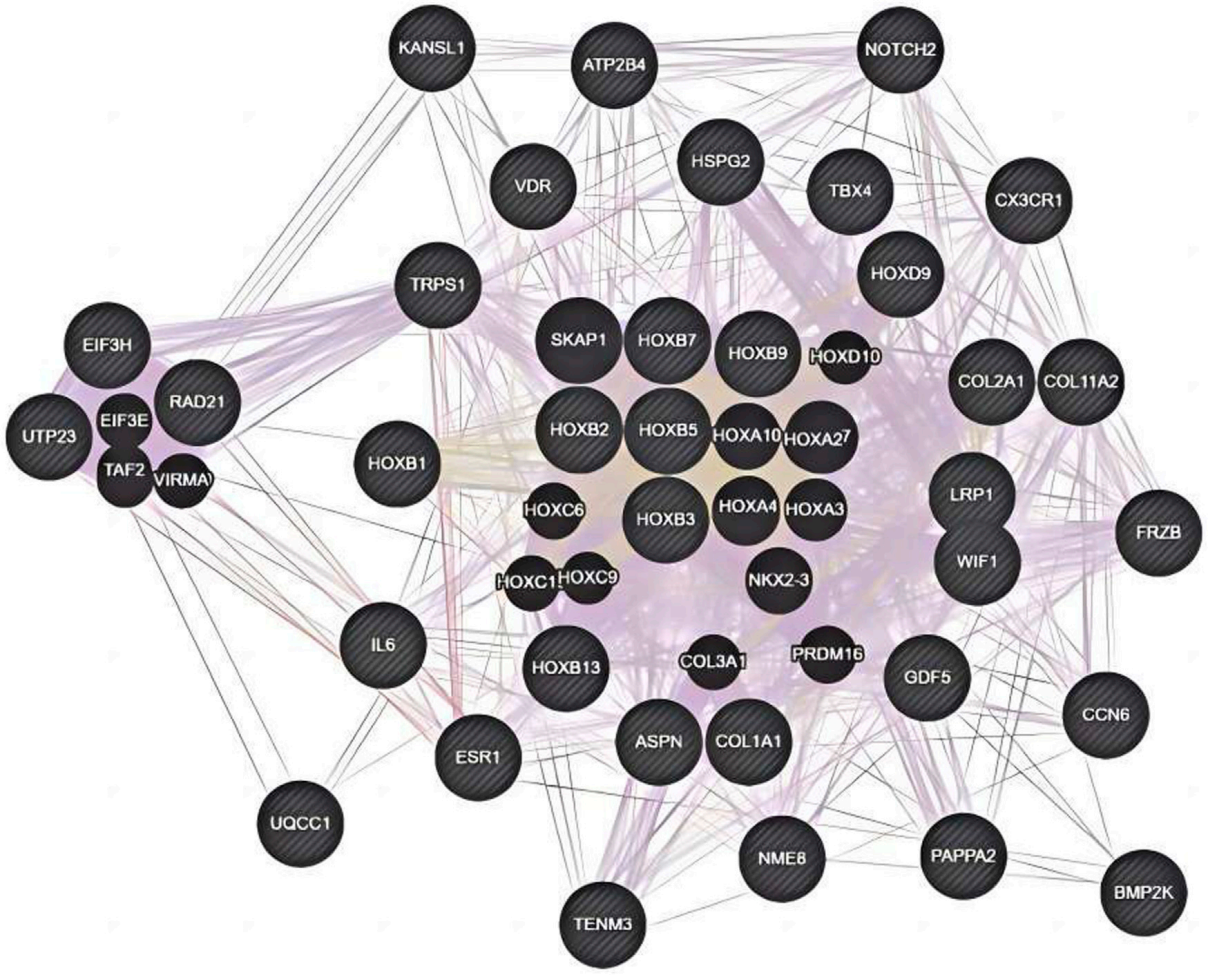
Co-expression, pathways, genetic interactions, physical interactions, shared protein domains for genes from Table 2 analyzed by Genemania software.

**FIGURE 2:**
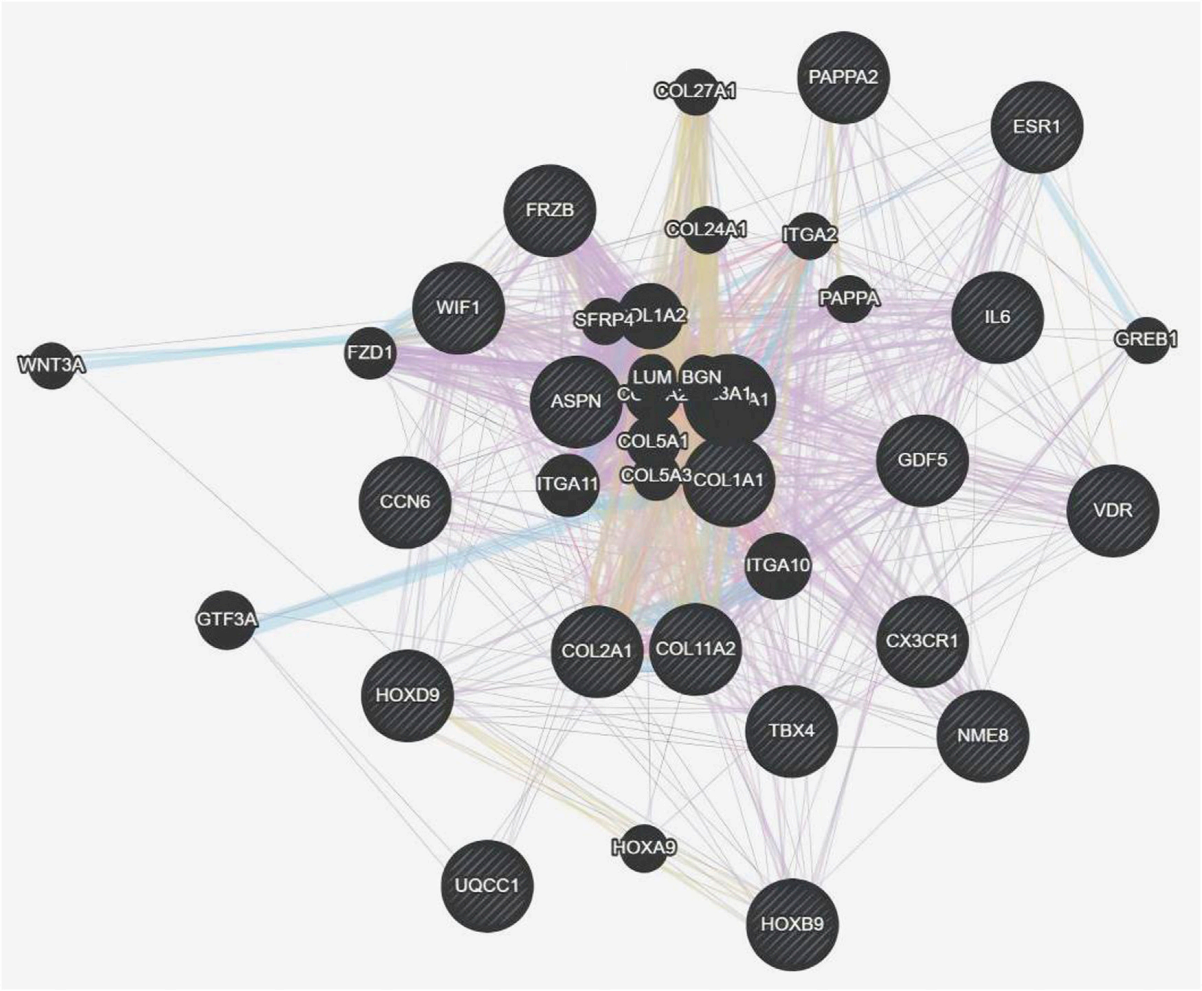
Co-expression, pathways, genetic interactions, physical interactions, shared protein domains for genes from Table 1 and Table 2 analyzed by Genemania software.

The genetic pattern of type II DDH, which is autosomal inheritance, is less prevalent. Feldman et al. reported a four-generation family in Utah with a variable phenotype (Sun et al., 2017). The gene variants screened from familial genetic studies or detected in sporadic cases were probably pathogenic genes, some of which were further explored in case-control studies. The variant *TXNDC3* rs10250905 was verified to be significantly associated with DDH both in 15 members of seven families and in case-control pairs consisting of 984 DDH children and 2043 healthy controls (Qiao et al., 2017). The variant *CX3CR1* rs3732378 screened using WES in four DDH members of an American family has been demonstrated to increase the risk of DDH by 12 times in the covert group and 75 times in the overt group in the Turkish population (Feldman et al., 2013; Gumus et al., 2021) However, the results are not always consistent. The *HSPG2* (c.3328G > T) and *ATP2B4* (c.2264G > A) variants detected by WES in five members of a Saudi family were not associated with the susceptibility to DDH in the Chinese Han population (Basit et al., 2017; Xu et al., 2021). This result indicates that the genes inherited in DDH families may be different from those in sporadic cases and may play a prominent genetic role. These findings may have been influenced by the small number of cases, the heterogeneity of DDH in pathogenic genes, and racial disparities. These findings lay the foundation for an early and accurate diagnosis of DDH in the future. Therefore, we analyzed the co-expression, pathways, genetic interactions, physical interactions, shared protein domains of DDH related genes concluded in Tables 1 and 2 (Figures 1, 2), which has shown the potential reaction of them. Futhermore, enreiched pathways of these genes analyzed in String database were shown in Table 4, suggesting the potential role of *COL2A1*, *COL11A2*, and *COL1A1* in the pathogensis of DHH. Meanwhile, it also suggested that DDH is probably caused by a combination of genes and can subsequently be affected by epigenetic, mechanical, or environmental factors (Zhang et al., 2020). In general, the genetic information from the DDH families and sporadic cases will enhance our understanding of DDH gene variants. Additional research with larger sample sizes could be highly valuable in terms of acquiring more information.

**TABLE 4:** Pathway enriched terms for gene variants in Tables 1 and 2.

ID	Description	Gene ratio	Genes	Strength	*p*-Value
8874081	MET activates PTK2 signaling	3/30	*COL2A1/COL11A2/COL1A1*	1.86	0.000179
8948216	Collagen chain trimerization	3/44	*COL2A1/COL11A2/COL1A1*	1.7	0.000179
30000171	Non-integrin membrane-ECM interactions	3/59	*COL2A1/COL11A2/COL1A1*	1.57	0.000179
2022090	Assembly of collagen fibrils and other multimeric structures	3/61	*COL2A1/COL11A2/COL1A1*	1.55	0.000179
1442490	Collagen degradation	3/64	*COL2A1/COL11A2/COL1A1*	1.53	0.000179
3000178	ECM proteoglycans	3/76	*COL2A1/ASPN/COL1A1*	1.46	0.000107
1474244	Extracellular matrix organization	5/300	*COL2A1/COL11A2/COL1A1/GDF5/ASPN*	1.08	8.14e-11

COL2A1, Collagen Type II Alpha 1 Chain; COL11A2, Collagen type XI alpha 2 chain; COL1A1, Collagen Type IAlpha 1 Chain; ASPN, Asporin; GDF5, Growth differentiation factor 5.

Gene variants detected in patients with DDH-related syndrome also contributed to the increasing number of DDH susceptibility genes. In the 18q deletion syndrome, which includes bilateral DDH, *HSPG2* variants were observed. Additionally, five members of the Saudi family were affected, despite the presence of distinct mutant loci (Basit et al., 2017; Yu et al., 2022). Ayoub et al. reported total or partial loss of *COL1A1* exon 6 in patients with EDS type arthrochalasia (aEDS) in 2019 (Ayoub et al., 2020). Additionally, a higher overall variation rate of the COL1A1 gene in Chinese female DDH patients was reported 6 years previously (Zhao et al., 2013a) The application of NGS (such as WES and ES) to DDH-related syndromes has the potential to produce more precise results. This will enable the establishment of connections between DDH and other clinical presentations in syndromes as well as the pursuit of an understanding of the pathogenesis of DDH from the perspective of mutation-related genetics.Notably, although DDH has been suggested to be annual screened in trisomy 21 patient (van Gijzen et al., 2019), there is still blank in the genetic study of trisomy 21 children with DDH.

Although accumulating data has demonstrated that genetic components play a crucial role in the etiology of DDH, most studies examining the pathophysiologic mechanisms underlying the development of DDH are superficial. The genetic basis of DDH remains largely unknown, and researchers recommend that further studies on the molecular mechanism of DDH be carried out based on variant genes and loci.

The future of DDH treatment is early screening, diagnosis, and treatment, which can prevent or reduce the occurrence of DDH with appropriate genetic counseling. Without reliable genetic data, early treatment cannot be effectively implemented. Understanding DDH associated genetics will aid in early disease diagnosis, which is essential for proper disease management and prevention through genetic counseling.

## Conclusion

There is a paucity of definitive data regarding the genetics of DDH. In this review, we highlighted the potentially crucial role of specific molecules in the pathogenesis of DDH, such as *CX3CR1*, *KANSL1*, *GDF5*, *TGFβ1*, *HSPG2*, and *COL1A1*, which merit further investigation. The data obtained by whole gene studies is beneficial for improving the accuracy of diagnosis when combined with clinical presentation and auxiliary detection. This leads to improved treatment efficiency and a reduced risk of movement impairment. However, it is important not to overlook the interindividual heterogeneity and the influence of environmental factors.

## References

[B1] Agenbag G. Vorster A. Julius S. Ramesar R. Beighton P. (2020). Namaqualand hip dysplasia in South Africa: the molecular determinant elucidated. S Afr. Med. J. 111 (1), 57–60. 10.7196/SAMJ.2020.v111i1.14561 33404007

[B2] Almuqbil M. AbuMelha A. Albokhari D. (2022). Milder presentation of autosomal dominant fatty acyl CoA reductase 1-related syndrome: report of the first Middle Eastern patient and review of the literature. Clin. Case Rep. 10 (10), e6307. 10.1002/ccr3.6307 36254151 PMC9562384

[B3] Al-Rakan M. A. Abothnain M. D. Alrifai M. T. Alfadhel M. (2018). Extending the ophthalmological phenotype of Galloway-Mowat syndrome with distinct retinal dysfunction: a report and review of ocular findings. BMC Ophthalmol. 18 (1), 147. 10.1186/s12886-018-0820-4 29929488 PMC6013877

[B4] Arenzana-Seisdedos F. Parmentier M. (2006). Genetics of resistance to HIV infection: role of co-receptors and co-receptor ligands. Semin. Immunol. 18 (6), 387–403. 10.1016/j.smim.2006.07.007 16978874

[B5] Ayoub S. Ghali N. Angwin C. Baker D. Baffini S. Brady A. F. (2020). Clinical features, molecular results, and management of 12 individuals with the rare arthrochalasia Ehlers-Danlos syndrome. Am. J. Med. Genet. A 182 (5), 994–1007. 10.1002/ajmg.a.61523 32091183

[B6] Baghdadi T. Nejadhosseinian M. Shirkoohi R. Mostafavi Tabatabaee R. Tamehri S. S. Saffari M. (2019). DNA hypermethylation of GDF5 in developmental dysplasia of the hip (DDH). Mol. Genet. Genomic Med. 7 (9), e887. 10.1002/mgg3.887 31338995 PMC6732267

[B7] Basit S. Albalawi A. M. Alharby E. Khoshhal K. I. (2017). Exome sequencing identified rare variants in genes HSPG2 and ATP2B4 in a family segregating developmental dysplasia of the hip. BMC Med. Genet. 18 (1), 34. 10.1186/s12881-017-0393-8 28327142 PMC5361705

[B8] Basit S. Alharby E. Albalawi A. M. Khoshhal K. I. (2018). Whole genome SNP genotyping in a family segregating developmental dysplasia of the hip detected runs of homozygosity on chromosomes 15q13.3 and 19p13.2. Congenit. Anom. (Kyoto) 58 (2), 56–61. 10.1111/cga.12235 28693051

[B9] Bauge C. Girard N. Lhuissier E. Bazille C. Boumediene K. (2014). Regulation and role of TGFβ signaling pathway in aging and osteoarthritis joints. Aging Dis. 5 (6), 394–405. 10.14336/AD.2014.0500394 25489490 PMC4249809

[B10] Bennet G. C. Rang M. Roye D. P. Aprin H. (1982). Dislocation of the hip in trisomy 21. J. Bone Jt. Surg. Br. 64 (3), 289–294. 10.1302/0301-620X.64B3.6212586 6212586

[B11] Bo N. Peng W. Xinghong P. Ma R. (2012). Early cartilage degeneration in a rat experimental model of developmental dysplasia of the hip. Connect. Tissue Res. 53 (6), 513–520. 10.3109/03008207.2012.700346 22670655

[B12] Cahill T. J. Sun X. Ravaud C. Villa Del Campo C. Klaourakis K. Lupu I. E. (2021). Tissue-resident macrophages regulate lymphatic vessel growth and patterning in the developing heart. Development 148 (3), dev194563. 10.1242/dev.194563 33462113 PMC7875498

[B13] Cengic T. Trkulja V. Pavelic S. K. Ratkaj I. Markova-Car E. Mikolaucic M. (2015). Association of TGFB1 29C/T and IL6 -572G/C polymorphisms with developmental hip dysplasia: a case-control study in adults with severe osteoarthritis. Int. Orthop. 39 (4), 793–798. 10.1007/s00264-015-2675-0 25603974

[B14] Chen C. H. Behringer R. R. (2022). Transgenic human HOXB1-9 directs anterior-posterior axial skeleton pattern in Hoxb1-9 deficient mice. Differentiation 127, 1–11. 10.1016/j.diff.2022.07.002 36041259

[B15] Chen J. Zhang W. B. He J. Z. Zhang R. Cao Y. Q. Liu X. (2018). Developmental dysplasia of the hip: a special pathology. Chin. J. Traumatol. 21 (4), 238–242. 10.1016/j.cjtee.2018.02.001 30007532 PMC6085197

[B16] Chen Y. Li L. Wang E. Zhang L. Zhao Q. (2019). Abnormal expression of Pappa2 gene may indirectly affect mouse hip development through the IGF signaling pathway. Endocrine 65 (2), 440–450. 10.1007/s12020-019-01975-0 31168749

[B17] Cheng B. Jia Y. Wen Y. Hou W. Xu K. Liang C. (2021). Integrative analysis of MicroRNA and mRNA sequencing data identifies novel candidate genes and pathways for developmental dysplasia of hip. Cartilage 13 (2_Suppl. l), 1618S–26S. 10.1177/1947603521990859 33522290 PMC8804775

[B18] Chijimatsu R. Saito T. (2019). Mechanisms of synovial joint and articular cartilage development. Cell Mol. Life Sci. 76 (20), 3939–3952. 10.1007/s00018-019-03191-5 31201464 PMC11105481

[B19] Christians J. K. de Zwaan D. R. Fung S. H. (2013). Pregnancy associated plasma protein A2 (PAPP-A2) affects bone size and shape and contributes to natural variation in postnatal growth in mice. PLoS One 8 (2), e56260. 10.1371/journal.pone.0056260 23457539 PMC3574143

[B20] Church V. L. Francis-West P. (2002). Wnt signalling during limb development. Int. J. Dev. Biol. 46 (7), 927–936. 12455630

[B21] Conover C. A. Boldt H. B. Bale L. K. Clifton K. B. Grell J. A. Mader J. R. (2011). Pregnancy-associated plasma protein-A2 (PAPP-A2): tissue expression and biological consequences of gene knockout in mice. Endocrinology 152 (7), 2837–2844. 10.1210/en.2011-0036 21586553

[B22] Cui R. R. Huang J. Yi L. Xie H. Zhou H. D. Yuan L. Q. (2007). WISP3 suppresses insulin-like growth factor signaling in human chondrocytes. Mol. Cell Endocrinol. 279 (1-2), 1–8. 10.1016/j.mce.2007.08.007 17942216

[B23] Dai J. Shi D. Zhu P. Qin J. Ni H. Xu Y. (2008). Association of a single nucleotide polymorphism in growth differentiate factor 5 with congenital dysplasia of the hip: a case-control study. Arthritis Res. Ther. 10 (5), R126. 10.1186/ar2540 18947434 PMC2592816

[B24] Del Brío Castillo R. Squires J. E. McKiernan P. J. (2019). A novel mutation in VPS33B gene causing a milder ARC syndrome phenotype with prolonged survival. JIMD Rep. 47 (1), 4–8. 10.1002/jmd2.12027 31240160 PMC6498830

[B25] Desteli E. E. Piskin A. Gulman A. B. Kaymaz F. Koksal B. Erdogan M. (2013). Estrogen receptors in hip joint capsule and ligamentum capitis femoris of babies with developmental dysplasia of the hip. Acta Orthop. Traumatol. Turc 47 (3), 158–161. 10.3944/aott.2013.2772 23748614

[B26] Ding R. Liu X. Zhang J. Yuan J. Zheng S. Cheng X. (2021). Downregulation of miR-1-3p expression inhibits the hypertrophy and mineralization of chondrocytes in DDH. J. Orthop. Surg. Res. 16 (1), 512. 10.1186/s13018-021-02666-1 34407854 PMC8371903

[B27] Doncheva N. T. Palasca O. Yarani R. Litman T. Anthon C. Groenen M. A. M. (2021). Human pathways in animal models: possibilities and limitations. Nucleic Acids Res. 49 (4), 1859–1871. 10.1093/nar/gkab012 33524155 PMC7913694

[B28] Dunn P. M. (1976). Perinatal observations on the etiology of congenital dislocation of the hip. Clin. Orthop. Relat. Res. 119, 11–22. 10.1097/00003086-197609000-00004 954299

[B29] Eftekhari H. Hosseini S. R. Pourreza Baboli H. Mafi Golchin M. Heidari L. Abedian Z. (2018). Association of interleukin-6 (rs1800796) but not transforming growth factor beta 1 (rs1800469) with serum calcium levels in osteoporotic patients. Gene 671, 21–27. 10.1016/j.gene.2018.05.118 29860063

[B30] Farquharson C. Lester D. Seawright E. Jefferies D. Houston B. (1999). Microtubules are potential regulators of growth-plate chondrocyte differentiation and hypertrophy. Bone 25 (4), 405–412. 10.1016/s8756-3282(99)00187-8 10511106

[B31] Feldman G. Dalsey C. Fertala K. Azimi D. Fortina P. Devoto M. (2010). The Otto Aufranc Award: identification of a 4 Mb region on chromosome 17q21 linked to developmental dysplasia of the hip in one 18-member, multigeneration family. Clin. Orthop. Relat. Res. 468 (2), 337–344. 10.1007/s11999-009-1073-6 19756907 PMC2807013

[B32] Feldman G. Kappes D. Mookerjee-Basu J. Freeman T. Fertala A. Parvizi J. (2019). Novel mutation in Teneurin 3 found to co-segregate in all affecteds in a multi-generation family with developmental dysplasia of the hip. J. Orthop. Res. 37 (1), 171–180. 10.1002/jor.24148 30273960

[B33] Feldman G. Offemaria A. Sawan H. Parvizi J. Freeman T. A. (2017). A murine model for developmental dysplasia of the hip: ablation of CX3CR1 affects acetabular morphology and gait. J. Transl. Med. 15 (1), 233. 10.1186/s12967-017-1335-0 29126427 PMC5681830

[B34] Feldman G. J. Parvizi J. Levenstien M. Scott K. Erickson J. A. Fortina P. (2013). Developmental dysplasia of the hip: linkage mapping and whole exome sequencing identify a shared variant in CX3CR1 in all affected members of a large multigeneration family. J. Bone Min. Res. 28 (12), 2540–2549. 10.1002/jbmr.1999 23716478

[B35] Feng W. J. Wang H. Shen C. Zhu J. F. Chen X. D. (2017). Severe cartilage degeneration in patients with developmental dysplasia of the hip. IUBMB Life 69 (3), 179–187. 10.1002/iub.1606 28185391

[B36] Frigola G. Del Rincon O. G. Florian V. B. Fita A. V. Campos B. Pauta M. (2021). Histopathology of recurrent Steel syndrome in fetuses caused by novel variants of COL27A1 gene. Virchows Arch. 479 (2), 413–418. 10.1007/s00428-020-02979-2 33411029

[B37] Funato N. Nakamura M. Richardson J. A. Srivastava D. Yanagisawa H. (2015). Loss of Tbx1 induces bone phenotypes similar to cleidocranial dysplasia. Hum. Mol. Genet. 24 (2), 424–435. 10.1093/hmg/ddu458 25209980

[B38] Gamerdinger U. Bosse K. Eggermann T. Kalscheuer V. Schwanitz G. Engels H. (2006). First report of a partial trisomy 3q12-q23 *de novo*--FISH breakpoint determination and phenotypic characterization. Eur. J. Med. Genet. 49 (3), 225–234. 10.1016/j.ejmg.2005.07.002 16762824

[B39] Genovesi M. L. Guadagnolo D. Marchionni E. Giovannetti A. Traversa A. Panzironi N. (2021). GDF5 mutation case report and a systematic review of molecular and clinical spectrum: expanding current knowledge on genotype-phenotype correlations. Bone 144, 115803. 10.1016/j.bone.2020.115803 33333243

[B40] Gkiatas I. Boptsi A. Tserga D. Gelalis I. Kosmas D. Pakos E. (2019). Developmental dysplasia of the hip: a systematic literature review of the genes related with its occurrence. EFORT Open Rev. 4 (10), 595–601. 10.1302/2058-5241.4.190006 31754465 PMC6836073

[B41] Gonzaga-Jauregui C. Yesil G. Nistala H. Gezdirici A. Bayram Y. Nannuru K. C. (2020). Functional biology of the Steel syndrome founder allele and evidence for clan genomics derivation of COL27A1 pathogenic alleles worldwide. Eur. J. Hum. Genet. 28 (9), 1243–1264. 10.1038/s41431-020-0632-x 32376988 PMC7608441

[B42] Granchi D. Stea S. Sudanese A. Toni A. Baldini N. Giunti A. (2002). Association of two gene polymorphisms with osteoarthritis secondary to hip dysplasia. Clin. Orthop. Relat. Res. 403 (403), 108–117. 10.1097/00003086-200210000-00018 12360016

[B43] Gumus E. Temiz E. Sarikaya B. Yuksekdag O. Sipahioglu S. Gonel A. (2021). The association between BMP-2, UQCC1 and CX3CR1 polymorphisms and the risk of developmental dysplasia of the hip. Indian J. Orthop. 55 (1), 169–175. 10.1007/s43465-020-00235-y 33569111 PMC7851229

[B44] Haider S. Lackner A. I. Dietrich B. Kunihs V. Haslinger P. Meinhardt G. (2022). Transforming growth factor-β signaling governs the differentiation program of extravillous trophoblasts in the developing human placenta. Proc. Natl. Acad. Sci. U. S. A. 119 (28), e2120667119. 10.1073/pnas.2120667119 35867736 PMC9282384

[B45] Han S. Xu X. Wen J. Wang J. Xiao S. Pan L. (2022). New genetic mutations in a Chinese child with Ehlers-Danlos syndrome-like spondyloepimetaphyseal dysplasia: a case report. Front. Pediatr. 10, 1073748. 10.3389/fped.2022.1073748 36619506 PMC9811192

[B46] Hao Z. Dai J. Shi D. Xu Z. Chen D. Zhao B. (2014). Association of a single nucleotide polymorphism in HOXB9 with developmental dysplasia of the hip: a case-control study. J. Orthop. Res. 32 (2), 179–182. 10.1002/jor.22507 24600698

[B47] Harsanyi S. Zamborsky R. Krajciova L. Bohmer D. Kokavec M. Danisovic L. (2021a). Association analysis of GDF5 and contributing factors in developmental dysplasia of the hip in infants. Ortop. Traumatol. Rehabil. 23 (5), 335–339. 10.5604/01.3001.0015.4348 34734566

[B48] Harsanyi S. Zamborsky R. Krajciova L. Kokavec M. Danisovic L. (2020). Developmental dysplasia of the hip: a review of etiopathogenesis, risk factors, and genetic aspects. Med. Kaunas. 56 (4), 153. 10.3390/medicina56040153 PMC7230892 32244273

[B49] Harsanyi S. Zamborsky R. Krajciova L. Kokavec M. Danisovic L. (2021b). Genetic study of IL6, GDF5 and PAPPA2 in association with developmental dysplasia of the hip. Genes (Basel) 12 (7), 986. 10.3390/genes12070986 34203285 PMC8303839

[B50] Hassan M. H. Elsadek A. A. M. Mahmoud M. A. Elsadek B. E. M. (2022). Vitamin D receptor gene polymorphisms and risk of knee osteoarthritis: possible correlations with TNF-α, macrophage migration inhibitory factor, and 25-hydroxycholecalciferol status. Biochem. Genet. 60 (2), 611–628. 10.1007/s10528-021-10116-0 34370118

[B51] Hatzikotoulas K. Roposch A. Consortium DDHCC Shah K. M. Clark M. J. Bratherton S. (2018). Genome-wide association study of developmental dysplasia of the hip identifies an association with GDF5. Commun. Biol. 1, 56. 10.1038/s42003-018-0052-4 30273415 PMC6123669

[B52] Igrek S. Onay T. Akgulle A. H. Polat M. Guney A. I. Muratli H. H. (2021). The association of interleukin-6 (IL-6) -572G/C and transforming growth factor beta 1 (TGFB1) 29C/T single nucleotide polymorphisms (SNPs) with developmental dysplasia of the hip: a case control study. Acta Chir. Orthop. Traumatol. Cech 88 (5), 339–343. 10.55095/achot2021/050 34738892

[B53] Ikeda K. Horie-Inoue K. Inoue S. (2019). Functions of estrogen and estrogen receptor signaling on skeletal muscle. J. Steroid Biochem. Mol. Biol. 191, 105375. 10.1016/j.jsbmb.2019.105375 31067490

[B54] Jawadi A. H. Wakeel A. Tamimi W. Nasr A. Iqbal Z. Mashhour A. (2018). Association analysis between four vitamin D receptor gene polymorphisms and developmental dysplasia of the hip. J. Genet. 97 (4), 925–930. 10.1007/s12041-018-0984-y 30262704

[B55] Ji X. Liu T. Zhao S. Li J. Li L. Wang E. (2020). WISP-2, an upregulated gene in hip cartilage from the DDH model rats, induces chondrocyte apoptosis through PPARγ *in vitro* . FASEB J. 34 (4), 4904–4917. 10.1096/fj.201901915R 32058630

[B56] Jia J. Li L. Zhao Q. Zhang L. Ru J. Liu X. (2012). Association of a single nucleotide polymorphism in pregnancy-associated plasma protein-A2 with developmental dysplasia of the hip: a case-control study. Osteoarthr. Cartil. 20 (1), 60–63. 10.1016/j.joca.2011.10.004 22037112

[B57] Kany S. Vollrath J. T. Relja B. (2019). Cytokines in inflammatory disease. Int. J. Mol. Sci. 20 (23), 6008. 10.3390/ijms20236008 31795299 PMC6929211

[B58] Kapoor B. Dunlop C. Wynn-Jones C. Fryer A. A. Strange R. C. Maffulli N. (2007). Vitamin D and oestrogen receptor polymorphisms in developmental dysplasia of the hip and primary protrusio acetabuli--a preliminary study. J. Negat. Results Biomed. 6, 7. 10.1186/1477-5751-6-7 17598904 PMC1929123

[B59] Kenanidis E. Gkekas N. K. Karasmani A. Anagnostis P. Christofilopoulos P. Tsiridis E. (2020). Genetic predisposition to developmental dysplasia of the hip. J. Arthroplasty 35 (1), 291–300. 10.1016/j.arth.2019.08.031 31522852

[B60] Kiapour A. M. Cao J. Young M. Capellini T. D. (2018). The role of Gdf5 regulatory regions in development of hip morphology. PLoS One 13 (11), e0202785. 10.1371/journal.pone.0202785 30388100 PMC6214493

[B61] Kim H. J. Prasad V. Hyung S. W. Lee Z. H. Lee S. W. Bhargava A. (2012). Plasma membrane calcium ATPase regulates bone mass by fine-tuning osteoclast differentiation and survival. J. Cell Biol. 199 (7), 1145–1158. 10.1083/jcb.201204067 23266958 PMC3529522

[B62] Kimura M. Taketani T. Horie A. Isumi H. Sejima H. Yamaguchi S. (2006). Two Japanese families with hyperekplexia who have a Arg271Gln mutation in the glycine receptor alpha 1 subunit gene. Brain Dev. 28 (4), 228–231. 10.1016/j.braindev.2005.08.007 16478653

[B63] Ko J. M. Yoo Y. Seo J. Cho J. S. Choi M. Chae J. H. (2016). Wiedemann-steiner syndrome with 2 novel KMT2A mutations: variable severity in psychomotor development and musculoskeletal manifestation. J. Child Neurology 32 (2), 237–242. 10.1177/0883073816674095 27777327

[B64] Kohara Y. Haraguchi R. Kitazawa R. Kitazawa S. (2020). Knockdown of Lrp1 in RAW264 cells inhibits osteoclast differentiation and osteoclast-osteoblast interactions *in vitro* . Biochem. Biophys. Res. Commun. 523 (4), 961–965. 10.1016/j.bbrc.2020.01.065 31964526

[B65] Kolundzic R. Trkulja V. Mikolaucic M. Kolundzic M. J. Pavelic S. K. Pavelic K. (2011). Association of interleukin-6 and transforming growth factor-β1 gene polymorphisms with developmental hip dysplasia and severe adult hip osteoarthritis: a preliminary study. Cytokine 54 (2), 125–128. 10.1016/j.cyto.2011.02.004 21353594

[B66] Li D. Wang H. He J. Y. Wang C. L. Feng W. J. Shen C. (2019). Inflammatory and fibrosis infiltration in synovium associated with the progression in developmental dysplasia of the hip. Mol. Med. Rep. 19 (4), 2808–2816. 10.3892/mmr.2019.9910 30720141

[B67] Li J. Hua X. Jones A. C. Williams S. Jin Z. Fisher J. (2016). The influence of the representation of collagen fibre organisation on the cartilage contact mechanics of the hip joint. J. Biomech. 49 (9), 1679–1685. 10.1016/j.jbiomech.2016.03.050 27079623 PMC4894261

[B68] Liu J. Zhou W. Chen Y. Li L. (2021). Acetabular development and fate of inverted limbus in rabbits: experimental observation from an animal model. J. Orthop. Res. 39 (12), 2595–2603. 10.1002/jor.25005 33580529

[B69] Liu L. Zhao M. Xie Z. G. Liu J. Peng H. P. Pei Y. F. (2020a). Twelve new genomic loci associated with bone mineral density. Front. Endocrinol. (Lausanne) 11, 243. 10.3389/fendo.2020.00243 32390946 PMC7188784

[B70] Liu X. Deng X. Ding R. Cheng X. Jia J. (2020b). Chondrocyte suppression is mediated by miR-129-5p via GDF11/SMAD3 signaling in developmental dysplasia of the hip. J. Orthop. Res. 38 (12), 2559–2572. 10.1002/jor.24713 32396235

[B71] Liu Y. Zan L. Zhao S. Huang H. Li Y. Tang Z. (2010). Molecular cloning, expression and characterization of bovine UQCC and its association with body measurement traits. Mol. Cells 30 (5), 393–401. 10.1007/s10059-010-0129-5 20811810

[B72] Loder R. T. Shafer C. (2015). The demographics of developmental hip dysplasia in the Midwestern United States (Indiana). J. Child. Orthop. 9 (1), 93–98. 10.1007/s11832-015-0636-1 25690337 PMC4340845

[B73] Lories R. J. Peeters J. Bakker A. Tylzanowski P. Derese I. Schrooten J. (2007). Articular cartilage and biomechanical properties of the long bones in Frzb-knockout mice. Arthritis Rheum. 56 (12), 4095–4103. 10.1002/art.23137 18050203

[B74] Loughlin J. (2011). Knee osteoarthritis, lumbar-disc degeneration and developmental dysplasia of the hip--an emerging genetic overlap. Arthritis Res. Ther. 13 (2), 108. 10.1186/ar3291 21542882 PMC3132037

[B75] Lowe D. A. Lepori-Bui N. Fomin P. V. Sloofman L. G. Zhou X. Farach-Carson M. C. (2014). Deficiency in perlecan/HSPG2 during bone development enhances osteogenesis and decreases quality of adult bone in mice. Calcif. Tissue Int. 95 (1), 29–38. 10.1007/s00223-014-9859-2 24798737 PMC4137566

[B76] Ma W. Zha Z. Chen K. Chen H. Wu Y. Ma J. (2017). Genetic association study of common variants in TGFB1 and IL-6 with developmental dysplasia of the hip in Han Chinese population. Sci. Rep. 7 (1), 10287. 10.1038/s41598-017-11185-1 28860542 PMC5579245

[B77] Maas S. Shaw A. Bikker H. Hennekam R. C. M. (1993). “Trichorhinophalangeal syndrome,” in GeneReviews((R)). Adam M. P. Everman D. B. Mirzaa G. M. Pagon R. A. Wallace S. E. Bean L. J. H. (Seattle (WA).

[B78] Machol K. Jain M. Almannai M. Orand T. Lu J. T. Tran A. (2017). Corner fracture type spondylometaphyseal dysplasia: overlap with type II collagenopathies. Am. J. Med. Genet. A 173 (3), 733–739. 10.1002/ajmg.a.38059 27888646 PMC5315610

[B79] Marini J. C. Forlino A. Bachinger H. P. Bishop N. J. Byers P. H. Paepe A. (2017). Osteogenesis imperfecta. Nat. Rev. Dis. Prim. 3, 17052. 10.1038/nrdp.2017.52 28820180

[B80] Mark P. R. Murray S. A. Yang T. Eby A. Lai A. Lu D. (2022). Autosomal recessive LRP1-related syndrome featuring cardiopulmonary dysfunction, bone dysmorphology, and corneal clouding. Cold Spring Harb. Mol. Case Stud. 8 (6), a006169. 10.1101/mcs.a006169 36307211 PMC9632358

[B81] Masri A. Shboul M. Khasawneh A. Jadallah R. Almustafa A. Escande-Beillard N. (2020). Congenital insensitivity to pain with anhidrosis syndrome: a series from Jordan. Clin. Neurol. Neurosurg. 189, 105636. 10.1016/j.clineuro.2019.105636 31841741

[B82] Mayo J. L. Holden D. N. Barrow J. R. Bridgewater L. C. (2009). The transcription factor Lc-Maf participates in Col27a1 regulation during chondrocyte maturation. Exp. Cell Res. 315 (13), 2293–2300. 10.1016/j.yexcr.2009.04.020 19414009 PMC3212405

[B83] Meulenbelt I. Chapman K. Dieguez-Gonzalez R. Shi D. Tsezou A. Dai J. (2009). Large replication study and meta-analyses of DVWA as an osteoarthritis susceptibility locus in European and Asian populations. Hum. Mol. Genet. 18 (8), 1518–1523. 10.1093/hmg/ddp053 19181678

[B84] Miyamoto Y. Shi D. Nakajima M. Ozaki K. Sudo A. Kotani A. (2008). Common variants in DVWA on chromosome 3p24.3 are associated with susceptibility to knee osteoarthritis. Nat. Genet. 40 (8), 994–998. 10.1038/ng.176 18622395

[B85] Muthuirulan P. Zhao D. Young M. Richard D. Liu Z. Emami A. (2021). Joint disease-specificity at the regulatory base-pair level. Nat. Commun. 12 (1), 4161. 10.1038/s41467-021-24345-9 34230488 PMC8260791

[B86] Nejadhosseinian M. Haerian H. Shirkoohi R. Karami J. Mortazavi S. M. J. (2022). Evaluation of CX3CR1 gene DNA methylation in developmental dysplasia of the hip (DDH). J. Orthop. Surg. Res. 17 (1), 436. 10.1186/s13018-022-03324-w 36175906 PMC9523927

[B87] Ning B. Jin R. Wan L. Wang D. (2018). Cellular and molecular changes to chondrocytes in an *in vitro* model of developmental dysplasia of the hip-an experimental model of DDH with swaddling position. Mol. Med. Rep. 18 (4), 3873–3881. 10.3892/mmr.2018.9384 30106106 PMC6131662

[B88] Palotie A. Vaisanen P. Ott J. Ryhanen L. Elima K. Vikkula M. (1989). Predisposition to familial osteoarthrosis linked to type II collagen gene. Lancet. 1 (8644), 924–927. 10.1016/s0140-6736(89)92507-5 2565419

[B89] Parker M. J. Fryer A. E. Shears D. J. Lachlan K. L. McKee S. A. Magee A. C. (2015). *De novo*, heterozygous, loss-of-function mutations in SYNGAP1 cause a syndromic form of intellectual disability. Am. J. Med. Genet. A 167A (10), 2231–2237. 10.1002/ajmg.a.37189 26079862 PMC4744742

[B90] Qiao L. Yan W. Dai J. Yao Y. Chen D. Xu Z. (2017). A novel missense variant in TXNDC3 is associated with developmental dysplasia of the hip in Han Chinese population. Int. J. Clin. Exp. Pathol. 10 (10), 10483–10488. 31966386 PMC6965766

[B91] Rauhio A. Uusi-Rasi K. Kunnas T. Nikkari S. T. Kannus P. Sievanen H. (2011). Estrogen receptor-1 genotype is associated with bone structure in premenopausal obese women. Maturitas 68 (4), 362–367. 10.1016/j.maturitas.2010.12.006 21216543

[B92] Rosenkrantz J. L. Gaffney J. E. Roberts V. H. J. Carbone L. Chavez S. L. (2021). Transcriptomic analysis of primate placentas and novel rhesus trophoblast cell lines informs investigations of human placentation. BMC Biol. 19 (1), 127. 10.1186/s12915-021-01056-7 34154587 PMC8218487

[B93] Rouault K. Scotet V. Autret S. Gaucher F. Dubrana F. Tanguy D. (2009). Do HOXB9 and COL1A1 genes play a role in congenital dislocation of the hip? Study in a Caucasian population. Osteoarthr. Cartil. 17 (8), 1099–1105. 10.1016/j.joca.2008.12.012 19341834

[B94] Rouault K. Scotet V. Autret S. Gaucher F. Dubrana F. Tanguy D. (2010). Evidence of association between GDF5 polymorphisms and congenital dislocation of the hip in a Caucasian population. Osteoarthr. Cartil. 18 (9), 1144–1149. 10.1016/j.joca.2010.05.018 20633687

[B95] Rubin S. Agrawal A. Stegmaier J. Krief S. Felsenthal N. Svorai J. (2021). Application of 3D MAPs pipeline identifies the morphological sequence chondrocytes undergo and the regulatory role of GDF5 in this process. Nat. Commun. 12 (1), 5363. 10.1038/s41467-021-25714-0 34508093 PMC8433335

[B96] Rubini M. Cavallaro A. Calzolari E. Bighetti G. Sollazzo V. (2008). Exclusion of COL2A1 and VDR as developmental dysplasia of the hip genes. Clin. Orthop. Relat. Res. 466 (4), 878–883. 10.1007/s11999-008-0120-z 18288556 PMC2504671

[B97] Sauvegarde C. Paul D. Bridoux L. Jouneau A. Degrelle S. Hue I. (2016). Dynamic pattern of HOXB9 protein localization during oocyte maturation and early embryonic development in mammals. PLoS One 11 (10), e0165898. 10.1371/journal.pone.0165898 27798681 PMC5087947

[B98] Schaeffer E. K. Study Group I. Mulpuri K. (2018). Developmental dysplasia of the hip: addressing evidence gaps with a multicentre prospective international study. Med. J. Aust. 208 (8), 359–364. 10.5694/mja18.00154 29716513

[B99] Schulte-Merker S. van Eeden F. J. Halpern M. E. Kimmel C. B. Nusslein-Volhard C. (1994). No tail (ntl) is the zebrafish homologue of the mouse T (Brachyury) gene. Development 120 (4), 1009–1015. 10.1242/dev.120.4.1009 7600949

[B100] Sekimoto T. Ishii M. Emi M. Kurogi S. Funamoto T. Yonezawa Y. (2017). Copy number loss in the region of the ASPN gene in patients with acetabular dysplasia: ASPN CNV in acetabular dysplasia. Bone Jt. Res. 6 (7), 439–445. 10.1302/2046-3758.67.BJR-2016-0094.R1 PMC5539304 28747338

[B101] Shi D. Dai J. Zhu P. Qin J. Zhu L. Zhu H. (2011). Association of the D repeat polymorphism in the ASPN gene with developmental dysplasia of the hip: a case-control study in Han Chinese. Arthritis Res. Ther. 13 (1), R27. 10.1186/ar3252 21329514 PMC3241371

[B102] Shi D. Nakamura T. Nakajima M. Dai J. Qin J. Ni H. (2008). Association of single-nucleotide polymorphisms in RHOB and TXNDC3 with knee osteoarthritis susceptibility: two case-control studies in East Asian populations and a meta-analysis. Arthritis Res. Ther. 10 (3), R54. 10.1186/ar2423 18471322 PMC2483443

[B103] Shi D. Sun W. Xu X. Hao Z. Dai J. Xu Z. (2014). A replication study for the association of rs726252 in PAPPA2 with developmental dysplasia of the hip in Chinese Han population. Biomed. Res. Int. 2014, 979520. 10.1155/2014/979520 24672801 PMC3930137

[B104] Storm E. E. Huynh T. V. Copeland N. G. Jenkins N. A. Kingsley D. M. Lee S. J. (1994). Limb alterations in brachypodism mice due to mutations in a new member of the TGF beta-superfamily. Nature 368 (6472), 639–643. 10.1038/368639a0 8145850

[B105] Sun Y. Wang C. Hao Z. Dai J. Chen D. Xu Z. (2015). A common variant of ubiquinol-cytochrome c reductase complex is associated with DDH. PLoS One 10 (4), e0120212. 10.1371/journal.pone.0120212 25848760 PMC4388640

[B106] Sun Y. Wang F. Sun X. Wang X. Zhang L. Li Y. (2017). CX3CR1 regulates osteoarthrosis chondrocyte proliferation and apoptosis via Wnt/β-catenin signaling. Biomed. Pharmacother. 96, 1317–1323. 10.1016/j.biopha.2017.11.080 29217163

[B107] Sun Y. You Y. Dai K. Zhang J. Yan M. Zhang Y. (2019b). Genetic variant of WIF1 gene is functionally associated with developmental dysplasia of the hip in Han Chinese population. Sci. Rep. 9 (1), 285. 10.1038/s41598-018-36532-8 30670715 PMC6342943

[B108] Sun Y. You Y. Jiang W. Zhai Z. Dai K. (2019a). 3D-bioprinting a genetically inspired cartilage scaffold with GDF5-conjugated BMSC-laden hydrogel and polymer for cartilage repair. Theranostics 9 (23), 6949–6961. 10.7150/thno.38061 31660079 PMC6815949

[B109] Tachmazidou I. Hatzikotoulas K. Southam L. Esparza-Gordillo J. Haberland V. Zheng J. (2019). Identification of new therapeutic targets for osteoarthritis through genome-wide analyses of UK Biobank data. Nat. Genet. 51 (2), 230–236. 10.1038/s41588-018-0327-1 30664745 PMC6400267

[B110] Tang J. Liu T. Wen X. Zhou Z. Yan J. Gao J. (2021). Estrogen-related receptors: novel potential regulators of osteoarthritis pathogenesis. Mol. Med. 27 (1), 5. 10.1186/s10020-021-00270-x 33446092 PMC7809777

[B111] Tian W. Zhao L. Wang J. Suo P. Wang J. Cheng L. (2012). Association analysis between HOXD9 genes and the development of developmental dysplasia of the hip in Chinese female Han population. BMC Musculoskelet. Disord. 13, 59. 10.1186/1471-2474-13-59 22520331 PMC3404944

[B112] Tickle C. (2015). How the embryo makes a limb: determination, polarity and identity. J. Anat. 227 (4), 418–430. 10.1111/joa.12361 26249743 PMC4580101

[B113] Todhunter R. J. Garrison S. J. Jordan J. Hunter L. Castelhano M. G. Ash K. (2019). Gene expression in hip soft tissues in incipient canine hip dysplasia and osteoarthritis. J. Orthop. Res. 37 (2), 313–324. 10.1002/jor.24178 30450639

[B114] Topak D. Seyithanoglu M. Dogar F. Karadeniz A. A. Tanriverdi B. Ozan F. (2021). Are vitamin D and vitamin D receptor levels different in children with developmental dysplasia of the hip? J. Orthop. Surg. Res. 16 (1), 24. 10.1186/s13018-020-02162-y 33413534 PMC7791744

[B115] van Gijzen A. F. M. Rouers E. D. M. van Douveren FQMP Dieleman J. Hendriks J. G. E. Halbertsma F. J. J. (2019). Developmental dysplasia of the hip in children with Down syndrome: comparison of clinical and radiological examinations in a local cohort. Eur. J. Pediatr. 178 (4), 559–564. 10.1007/s00431-019-03322-x 30710155

[B116] Vaquero-Picado A. Gonzalez-Moran G. Garay E. G. Moraleda L. (2019). Developmental dysplasia of the hip: update of management. EFORT Open Rev. 4 (9), 548–556. 10.1302/2058-5241.4.180019 31598333 PMC6771078

[B117] Wang C. L. Zuo B. Li D. Zhu J. F. Xiao F. Zhang X. L. (2020). The long noncoding RNA H19 attenuates force-driven cartilage degeneration via miR-483-5p/Dusp5. Biochem. Biophys. Res. Commun. 529 (2), 210–217. 10.1016/j.bbrc.2020.05.180 32703413

[B118] Wang H. Zhang X. He J. Y. Zheng X. F. Li D. Li Z. (2015). Increasing expression of substance P and calcitonin gene-related peptide in synovial tissue and fluid contribute to the progress of arthritis in developmental dysplasia of the hip. Arthritis Res. Ther. 17 (1), 4. 10.1186/s13075-014-0513-1 25578529 PMC4320827

[B119] Wang H. Zheng X. F. Zhang X. Li Z. Shen C. Zhu J. F. (2014). Increasing substance P levels in serum and synovial tissues from patients with developmental dysplasia of the hip (DDH). BMC Musculoskelet. Disord. 15, 92. 10.1186/1471-2474-15-92 24642234 PMC3995111

[B120] Wang K. Shi D. Zhu P. Dai J. Zhu L. Zhu H. (2010). Association of a single nucleotide polymorphism in Tbx4 with developmental dysplasia of the hip: a case-control study. Osteoarthr. Cartil. 18 (12), 1592–1595. 10.1016/j.joca.2010.09.008 20887794

[B121] Weisfeld-Adams J. D. Edelmann L. Gadi I. K. Mehta L. (2012). Phenotypic heterogeneity in a family with a small atypical microduplication of chromosome 22q11.2 involving TBX1. Eur. J. Med. Genet. 55 (12), 732–736. 10.1016/j.ejmg.2012.08.011 23059467

[B122] Woodacre T. Ball T. Cox P. (2016). Epidemiology of developmental dysplasia of the hip within the UK: refining the risk factors. J. Child. Orthop. 10 (6), 633–642. 10.1007/s11832-016-0798-5 27866316 PMC5145848

[B123] Wu R. Gao G. Xu Y. (2020). Electrospun fibers immobilized with BMP-2 mediated by polydopamine combined with autogenous tendon to repair developmental dysplasia of the hip in a porcine model. Int. J. Nanomedicine 15, 6563–6577. 10.2147/IJN.S259028 32982218 PMC7490068

[B124] Xu J. Ye W. Li H. Xu L. (2022a). WNT1 expression influences the development of dysplasia of the hip via regulating RBPMS2/NOG-BMP2/4-GDF5- WISP2 pathway. Nucleosides Nucleotides Nucleic Acids 41 (8), 765–777. 10.1080/15257770.2022.2081337 35675541

[B125] Xu R. Jiang X. Lu J. Wang K. Sun Y. Zhang Y. (2020). Genetic variant of COL11A2 gene is functionally associated with developmental dysplasia of the hip in Chinese Han population. Aging (Albany NY) 12 (9), 7694–7703. 10.18632/aging.103040 32396528 PMC7244083

[B126] Xu R. Zhang F. Lu J. Wang K. Pan P. Sun Y. (2021b). Secreted frizzled-related protein 3 was genetically and functionally associated with developmental dysplasia of the hip. Aging (Albany NY) 13 (8), 11281–11295. 10.18632/aging.202815 33820867 PMC8109121

[B127] Xu X. Bi X. Wang J. Gui R. Li T. Li L. (2022b). Identification of KANSL1 as a novel pathogenic gene for developmental dysplasia of the hip. J. Mol. Med. Berlin, Ger. 100 (8), 1159–1168. 10.1007/s00109-022-02220-4 35727364

[B128] Xu X. Wang B. Chen Y. Zhou W. Li L. (2021a). Replicative verification of susceptibility genes previously identified from families with segregating developmental dysplasia of the hip. Ital. J. Pediatr. 47 (1), 140. 10.1186/s13052-021-01087-4 34174923 PMC8234666

[B129] Yamanaka M. Ishijima M. Tokita A. Sakamoto Y. Kaneko H. Maezawa K. (2009). Association of oestrogen receptor gene polymorphism with the long-term results of rotational acetabular osteotomy. Int. Orthop. 33 (4), 1155–1164. 10.1007/s00264-009-0730-4 19219433 PMC2898992

[B130] Yan W. Hao Z. Tang S. Dai J. Zheng L. Yu P. (2019). A genome-wide association study identifies new genes associated with developmental dysplasia of the hip. Clin. Genet. 95 (3), 345–355. 10.1111/cge.13483 30511388

[B131] Yan W. Zheng L. Xu X. Hao Z. Zhang Y. Lu J. (2022). Heterozygous LRP1 deficiency causes developmental dysplasia of the hip by impairing triradiate chondrocytes differentiation due to inhibition of autophagy. Proc. Natl. Acad. Sci. U. S. A. 119 (37), e2203557119. 10.1073/pnas.2203557119 36067312 PMC9477389

[B132] Yang S. Zusman N. Lieberman E. Goldstein R. Y. (2019). Developmental dysplasia of the hip. Pediatrics 143 (1), e20181147. 10.1542/peds.2018-1147 30587534

[B133] Yang W. Jin G. Qian K. Zhang C. Zhi W. Yang D. (2022). Comprehensive bioinformatics analysis of susceptibility genes for developmental dysplasia of the hip. Intractable Rare Dis. Res. 11 (2), 70–80. 10.5582/irdr.2022.01043 35702583 PMC9161127

[B134] Yerges-Armstrong L. M. Yau M. S. Liu Y. Krishnan S. Renner J. B. Eaton C. B. (2014). Association analysis of BMD-associated SNPs with knee osteoarthritis. J. Bone Min. Res. 29 (6), 1373–1379. 10.1002/jbmr.2160 PMC4080308 24339167

[B135] Yu S. Wang C. Lei K. Leng X. Zhang L. Tian F. (2022). Case report: genetic analysis of a child with 18q deletion syndrome and developmental dysplasia of the hip. BMC Med. Genomics 15 (1), 199. 10.1186/s12920-022-01345-2 36123715 PMC9484224

[B136] Zamborsky R. Kokavec M. Harsanyi S. Attia D. Danisovic L. (2019). Developmental dysplasia of hip: perspectives in genetic screening. Med. Sci. (Basel). 7 (4), 59. 10.3390/medsci7040059 30979092 PMC6524033

[B137] Zhang C. H. Gao Y. Hung H. H. Zhuo Z. Grodzinsky A. J. Lassar A. B. (2022). Creb5 coordinates synovial joint formation with the genesis of articular cartilage. Nat. Commun. 13 (1), 7295. 10.1038/s41467-022-35010-0 36435829 PMC9701237

[B138] Zhang J. Yan M. Zhang Y. Yang H. Sun Y. (2018). Association analysis on polymorphisms in WISP3 gene and developmental dysplasia of the hip in Han Chinese population: a case-control study. Gene 664, 192–195. 10.1016/j.gene.2018.04.020 29680248

[B139] Zhang L. Xu X. Chen Y. Li L. Zhang L. Li Q. (2020a). Mapping of developmental dysplasia of the hip to two novel regions at 8q23-q24 and 12p12. Exp. Ther. Med. 19 (4), 2799–2803. 10.3892/etm.2020.8513 32256763 PMC7086185

[B140] Zhang S. Doudoulakis K. J. Khurwal A. Sarraf K. M. (2020b). Developmental dysplasia of the hip. Br. J. Hosp. Med. (Lond) 81 (7), 1–8. 10.12968/hmed.2020.0223 32730146

[B141] Zhang X. Meng Q. Ma R. Chen G. Cheng L. Shen J. (2015). Early acetabular cartilage degeneration in a rabbit model of developmental dysplasia of the hip. Int. J. Clin. Exp. Med. 8 (8), 14505–14512. 26550441 PMC4613126

[B142] Zhao L. Ma Q. Feng X. Fan L. Jiao Q. Wang S. (2019). Screening for developmental dysplasia of the hip in infants in tibet identifies increased prevalence associated with altitude. Med. Sci. Monit. 25, 5771–5775. 10.12659/MSM.916456 31376279 PMC6690215

[B143] Zhao L. Pan H. Wang J. Cheng Z. Cheng L. Wang B. (2013b). Two single nucleotide polymorphisms in the GDF5 gene are associated with development dysplasia of the hip in Chinese female population. Sci. China Life Sci. 56 (11), 1063–1065. 10.1007/s11427-013-4514-0 24114442

[B144] Zhao L. Tian W. Pan H. Zhu X. Wang J. Cheng Z. (2013a). Variations of the COL1A1 gene promoter and the relation to developmental dysplasia of the hip. Genet. Test. Mol. Biomarkers 17 (11), 840–843. 10.1089/gtmb.2013.0179 23941072

[B145] Zhao L. Zhou Z. Wang S. Jiao Q. Wu J. Ma F. (2017). A recurrent mutation in bone morphogenetic proteins-2-inducible kinase gene is associated with developmental dysplasia of the hip. Exp. Ther. Med. 13 (5), 1773–1778. 10.3892/etm.2017.4191 28565766 PMC5443164

[B146] Zhou W. Luo W. Liu D. Canavese F. Li L. Zhao Q. (2022). Fluoride increases the susceptibility of developmental dysplasia of the hip via increasing capsular laxity triggered by cell apoptosis and oxidative stress *in vivo* and *in vitro* . Ecotoxicol. Environ. Saf. 234, 113408. 10.1016/j.ecoenv.2022.113408 35298972

[B147] Zhu L. Shi D. Dai J. Qin J. Fan J. Wang Z. (2011). Lack of evidence for association between DVWA gene polymorphisms and developmental dysplasia of the hip in Chinese Han population. Rheumatol. Int. 31 (7), 883–887. 10.1007/s00296-010-1410-9 20238217

[B148] Zhu L. Q. Su G. H. Dai J. Zhang W. Y. Yin C. H. Zhang F. Y. (2019). Whole genome sequencing of pairwise human subjects reveals DNA mutations specific to developmental dysplasia of the hip. Genomics 111 (3), 320–326. 10.1016/j.ygeno.2018.02.006 29486210

[B149] Zieglgansberger W. (2019). Substance P and pain chronicity. Cell Tissue Res. 375 (1), 227–241. 10.1007/s00441-018-2922-y 30284083 PMC6335504

